# Insights into the Hierarchical Assembly of a Chemically Diverse Peptide Hydrogel Derived from Human Semenogelin I

**DOI:** 10.1021/acsnano.4c08672

**Published:** 2024-11-01

**Authors:** Brett H. Pogostin, Kerilyn Godbe, Marija Dubackic, Isabelle Angstman, William Fox, Natalie Giovino, Matija Lagator, Abigail Payson, Marisa LaBarca, Birgitta Frohm, Katja Bernfur, Sara Linse, Casey H. Londergan, Ulf Olsson, Luigi Gentile, Karin S. Åkerfeldt

**Affiliations:** † Department of Chemistry, Haverford College, Haverford, Pennsylvania 19041, United States; ‡ Department of Physical Chemistry, Lund University, PO Box 124, Lund SE-221 00, Sweden; § Biochemistry and Structural Biology, Lund University, PO Box 124, Lund SE-221 00, Sweden; ∥ Department of Chemistry, University of Bari Aldo Moro, Via Orabona 4, Bari 70126, Italy

**Keywords:** protein fragment, peptide aggregation, amyloid, self-assembly, X-ray scattering, supramolecular assembly, isotope effects

## Abstract

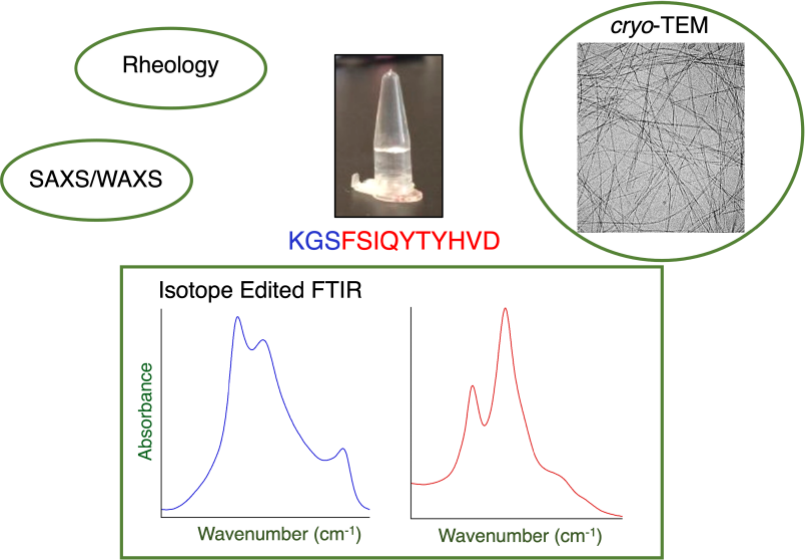

A peptide corresponding to a 13-residue segment of the human protein semenogelin I has been shown to generate a hydrogel consisting of amyloid-like fibrils. The relative chemical diversity (compared to synthetic *de novo* sequences) with 11 distinct amino acids makes this peptide (P0) an ideal candidate for investigating the role of individual residues in gelation. Herein, the *N-*terminal residues have been sequentially removed to furnish a series of truncated peptides, P1–P10, ranging from 12 to 3 residues in length. FTIR spectroscopy investigations reveal that P0–P6 forms a β-sheet secondary structure while shorter sequences do not self-assemble. Site-specific isotope labeling of the amide backbone of P0–P2 with the IR-sensitive vibrational probe ^13^C=O yields FTIR spectra indicative of the initial formation of a kinetic product that slowly transforms into a structurally different thermodynamic product. The effects of the isotopic labels on the IR spectra facilitate the assignment of parallel and antiparallel structures, which are sometimes coexistent. Additional IR studies of three Phe^CN^-labeled P0 sequences are consistent with an H-bonded β-sheet amide core, spanning the 7 central residues. The macromolecular assembly of peptides that form β-sheets was assessed by cryo*-*TEM, SAXS/WAXS, and rheology. Cryo*-*TEM images of peptides P1–P6 display μm-long nanofibrils. Peptides P0–P3 generate homogeneous hydrogels composed of colloidally stable nanofibrils, and P4–P6 undergo phase separation due to the accumulation of attractive interfibrillar interactions. Three amino acid residues, Ser39, Phe40, and Gln43, were identified to be of particular interest in the truncated peptide series as the removal of any one of them, as the sequence shortens, leads to a major change in material properties.

## 1 Introduction

Hydrogels are three-dimensional polymer matrices that can hold high quantities (>99%) of water within their structure.^1,2^ Biopolymer-based gels may consist of carbohydrates, peptides, and proteins, or oligonucleotides.^2−5^ Hydrogels of natural biopolymers can offer advantages over their synthetic counterparts due to their biocompatibility, low cytotoxicity, and biodegradability. They have been employed in various biomedical applications to promote cell adhesion, proliferation, and regeneration of tissue.^6^ Self-healing hydrogels, the focus of this work, may also be used as injectable biomaterials for the encapsulation and slow release of drugs.^7,8^

Peptide-based hydrogels are promising biomaterials due to their sequence modularity, allowing for the inclusion of bioactive motifs and targeted tuning of desired material properties.^9,10^ The chemical and physical properties of *de novo-* designed peptide hydrogels are adjustable by changing the peptide length and composition of amino acid building blocks, which can be chosen from a diverse library of natural and noncanonical residues. Examples of peptide hydrogels formed from *de novo* designed synthetic peptides include the minimalist β-hairpin MAX series,^11−14^ which are based on the parent amphiphilic 20-residue sequence H_2_N–VKVKVKVK–(V^D^PPT)–KVKVKVKV–CONH_2_. Additional simple repeat peptides that generate β-sheet-based fibrils include CH_3_O–(RADA)_4_–CONH_2_ (RADA16),^15^ and the multidomain peptide (MDP) hydrogel series, with the general structure CH_3_O–XX(SL)_6_XX–CONH_2_, where X is a charged amino acid such as Lys, Arg, Asp, or Glu.^16^ Other synthetically derived peptide-based hydrogel systems rely on an aliphatic tail to drive their self-assembly, such as peptide amphiphiles^17^ or helical peptides, including collagen mimetics.^18^ Some peptide hydrogels are derived from peptide sequences found in naturally occurring proteins. Examples of natural peptides and segments of proteins that form hydrogels include collagen,^19^ bovine serum albumin,^4,5,20^ α-synuclein,^21−24^ human islet polypeptide,^25^ a fragment of human cardiac troponin C,^26^ a peptide sequence from the coronavirus spike protein,^27^ and semenogelin.^28^

The aggregation of peptide monomers to generate a hydrogel follows a hierarchical buildup in which monomers, typically amphiphilic, associate and self-assemble into fibrils.^29^ The specific surface properties of the fibrils govern their higher-order assembly into a supramolecular, water-trapping, percolating gel network. The balance of attractive and repulsive electrostatic interactions between fibrils is key as excessive repulsions prevent fibril assembly while dominating attractive interactions lead to the generation of colloidally unstable aggregates.^24,30^ Further manipulation of the gel properties can be achieved by changing the conditions of the surrounding aqueous medium by modifying salinity, temperature, and pH or through the addition of specific additives, such as metal ions and chemical covalent cross-linkers.^16,24,31^ Many sequence structure–property investigations of peptide hydrogels have focused on synthetic sequences—like RADA16, MAX, and MDPs—or on short hydrogel-forming tri- and dipeptides.^5,32−34^ These *de novo* hydrogel systems, however, rely on repeating residue motifs and have limited intrasequence chemical diversity. Fewer studies have attempted to directly correlate amino acid content and sequence with material properties in hydrogels derived from chemically diverse, naturally occurring peptide sequences.

The present study targets a relatively complex 13-residue peptide for which we attempt to uncover the underlying chemical principles behind hydrogelation and correlations between sequence, structure, and bulk mechanical properties. The peptide is derived from the human protein semenogelin I (SgI), which is the main proteinaceous constituent in seminal plasma. SgI is also present in nonreproductive tissues including skeletal muscles, the trachea, and the gastrointestinal tract.^35^ We previously reported that a short fragment of SgI forms a pH-responsive gel, similar in properties to the full-length protein.^28^ The short *N*- and C-capped 11-residue peptide, containing residues 38–48 of the native protein (SgI_38–48_), AcNH–GSFSIQYTYHV–CONH_2_, self-assembles at low concentration (0.5% w/v) and under physiological conditions to generate a physically cross-linked polymer network capable of sequestering large amounts of water. This amphiphilic peptide is composed of alternating polar and nonpolar residues, which suggests a propensity to aggregate into β-sheet-rich fibrils, where the secondary structure was verified by Fourier-transform infrared spectroscopy (FTIR) and circular dichroism (CD) spectroscopy.^28^ The importance of this segment to the hydrogel formation of the full-length native protein is supported by the fact that the protease human prostate-specific antigen (PSA), responsible for breaking up the semen gel to release sperm, cleaves SgI at Y44.

**Figure 1:**
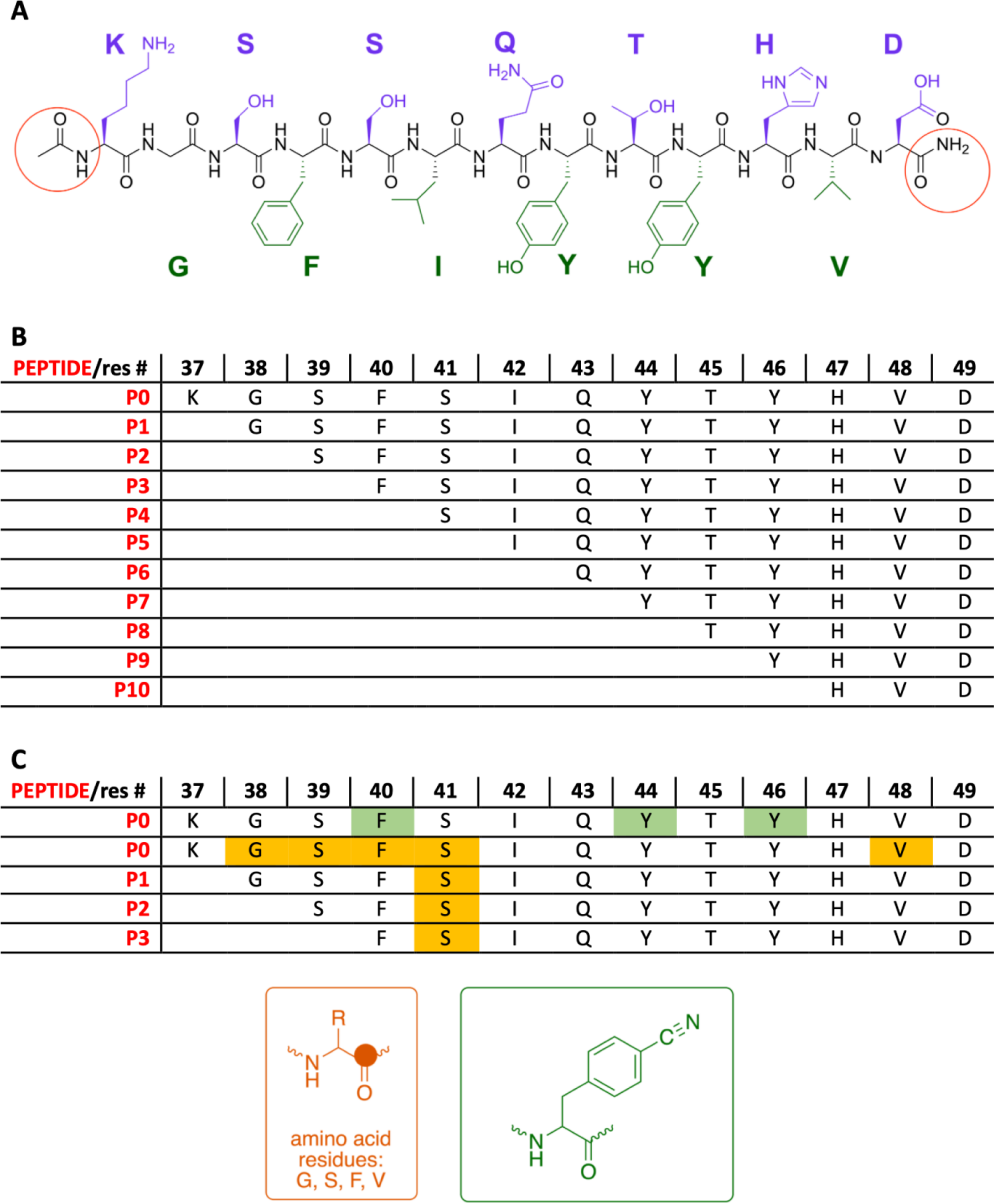
(A) Chemical structure of SgI_37–49_ (P0), corresponding to amino acid residues 37–49 of human semenogelin I, highlighting the alternating hydrophobic (green) and hydrophilic (purple) residues, with the neutral *N*-acetyl and C-amide end caps circled in red. (B) The sequences of peptides P0–P10 were investigated in this study. All of the peptides were similarly capped at both ends. (C) The residues in P0–P3, labeled with a vibrationally sensitive probe, one at a time, with a backbone amide ^13^C=^16^O (orange) or a Phe^CN^ (green), with the chemical structures of the probes shown in the orange (^13^C atom solid circle) and green boxes, respectively.

SgI_38–48_ is highly hydrophobic and displays a limited solubility in water, which makes this peptide difficult to handle and isolate. The naturally flanking K37 and D49 residues, which extend the polar/nonpolar pattern, were therefore added to create a 13-residue sequence, SgI_37–49_,^36,37^ also referred to as the “KD peptide,”^38,39^ and named peptide 0 (P0) in the present study (Figure 1A). To identify the residues that contribute to gelation and to find how the secondary structure relates to its materials properties, we studied a series of truncated sequences originating from P0 where one amino acid at a time was systematically removed from the *N*-terminus, comprising 12 residues (P1) down to 3 residues (P10) (Figure 1B). All peptides, including the shortest one, AcNH–HVD–CONH_2_, retain the histidine residue responsible for the pH-dependent aggregation behavior of P0.^37,40^ The peptides P0–P10, native and labeled (Figure 1C), were studied using FTIR spectroscopy, cryo-transmission electron microscopy (cryo*-*TEM), small- and wide-angle X-ray scattering (SAXS and WAXS), and rheology measurements, with the aim of investigating the interplay between peptide length, secondary structure, supramolecular assemblage, and material properties.

## 2 Results and Discussion

### 2.1 Secondary Structure

#### 2.1.1 FTIR Spectroscopy of Peptides P0–P8

All FTIR measurements were collected on samples where a trifluoroacetate (TFA^–^) to chloride ion exchange was performed to eliminate signals from the TFA^–^ carbonyl moiety in the amide I spectral region (Figure S1). FTIR spectra were collected for P0–P5 in D_2_O at six pD values (3.0, 5.0, 7.0, 8.0, 9.0, and 10.0) (Figures S2–S7). FTIR analysis for P6–P8 was performed at a pD of 8.0 (Figures S8–S10). Samples were also evaluated for gelation through visual inspection (images shown in Figures S2–S8, pD 8.0). The bulk of FTIR studies were performed at pD 8.0, as the optimum pH range for hydrogelation is 7–9 for both SgI_38–48_^28^ and P0.

**Figure 2:**
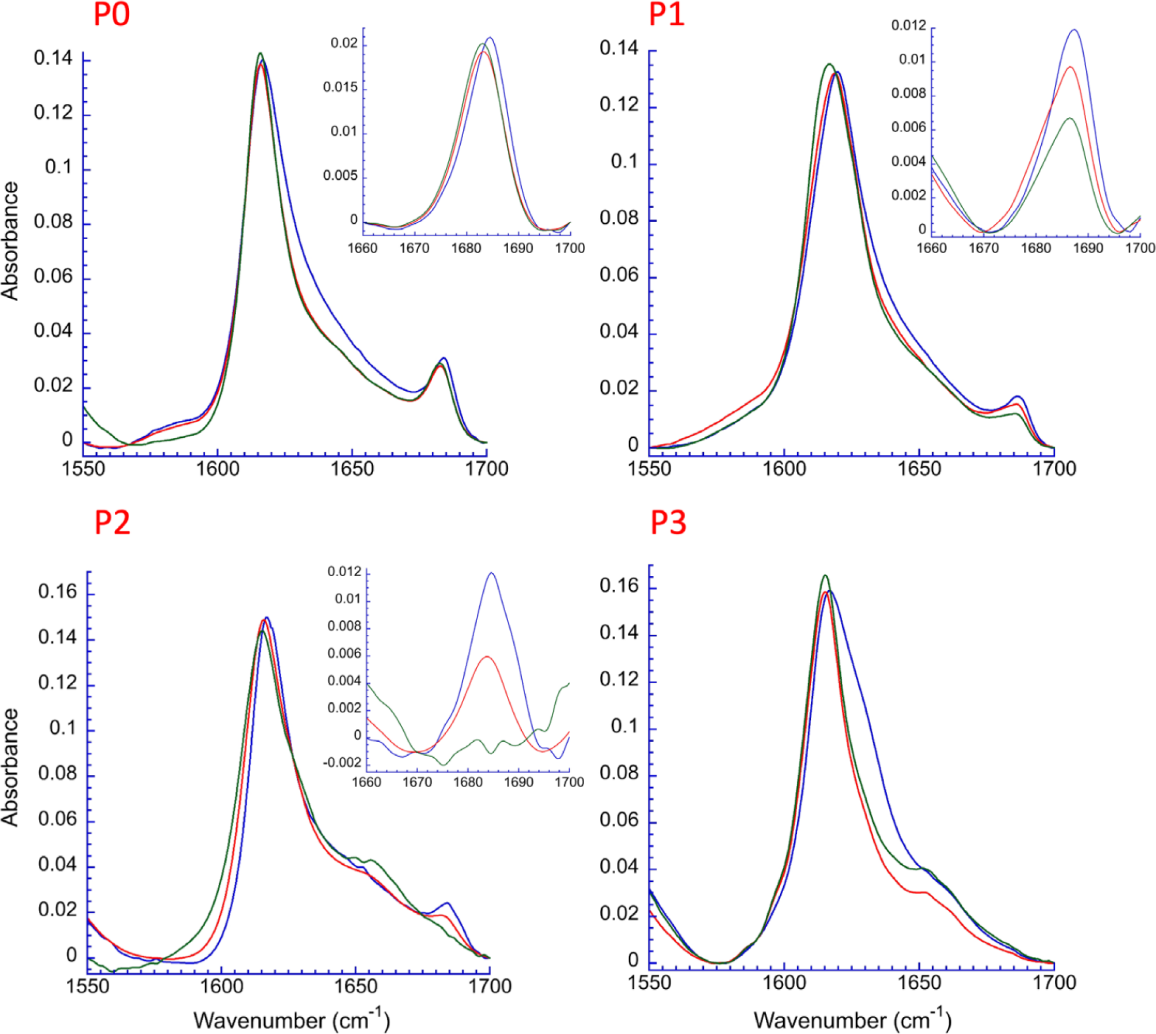
FTIR spectra of 2.0 mM P0, P1, P2, and P3 at time 0 h (blue), 48 h (red), and 1 week (green) at ambient temperature at pD 7.0. Signals are observed at ca. 1615–1619 and 1684 cm^–1^ consistent with β-sheet fibrils. Insets are shown for P0–P2, highlighting the high-frequency band area of 1660–1700 cm^–1^, which remains for P0, decreases in intensity with time for P1 and P2, and is absent for P3.

Figure 2 shows the amide I region of the FTIR spectra obtained for P0–P3 at pD 7.0, immediately after dissolution in D_2_O and pD adjustment (0 h), at 48 h and after 1 week of incubation at ambient temperature (spectra overlays for P0–P3 at pD 7.0 and 8.0 are also shown in Figures S11 and S12). The spectra for P0–P2 all display a strong and narrow signal centered at slightly below 1620 cm^–1^, indicative of a well-ordered β-sheet, with an associated weaker and narrow high-frequency amide I region band at 1685 cm^–1^.^41−47^ The data for P3, while lacking the 1685 cm^–1^ band, are also consistent with the β-sheet secondary structure, as indicated by the narrow and strong signal at 1620 cm^–1^. Similar to P3, at pD 8.0, the shorter peptides P4, P5, and P6 display the 1620 cm^–1^ band in addition to a signal at around 1650 cm^–1^, consistent with random coil content (Figures S6-S8). The spectra for P7 and P8 show no sign of a β-sheet secondary structure (Figures S9 and S10).

The narrow amide I signal at or below 1620 cm^–1^ is characteristic of an amyloid fibril-like β-sheet secondary structure, with extensive backbone dehydration and long-range molecular ordering within the sheet, in contrast to β-sheets in globular proteins, which typically show a broader band at a higher frequency around 1630–1635 cm^–1^.^48−50^ For P0, the amide I signal is seen at 1619 cm^–1^ with an associated lower intensity and higher frequency band at around 1685 cm^–1^, which is typically taken to be an indicator of an antiparallel arrangement within the β-sheet and may suggest a parallel arrangement when absent. This interpretation, however, should be taken with some caution since other types of structures, such as turns, might also result in signals in this high amide I frequency spectral region.^51^ With these precautions in mind, the simplest interpretation in our case is that P0, P1, and P2 generate fibrils consisting of well-ordered β-strands; however, the intensity of the high-frequency band appears to decrease with incubation time, particularly for P1 and P2, comparing data obtained at 48 h to 1 week (Figures 2, S11, and S12). The presence of this band also appears to be pD-dependent. For P0, it is present at all pD values tested but is less pronounced at pD values of 2.9 and 5.24 after 1 week (Figure S2). In the case of P1 and P2, the signal intensity also varies with the pD (Figures S3 and S4). These data suggest that P0–P2 fibrils may be prone to structural changes, which we further investigated using isotope-edited (IE) FTIR spectroscopy.^52,53^

#### 2.1.2 Secondary Structure Determination by Isotope-Edited FTIR

A series of 5 singly ^13^C=O-labeled P0 peptides were made, with the labeled residue marked *, at positions Gly38*, Ser39*, Phe40*, Ser41*, or Val48* (Figure 1C). The IE FTIR method is based on the sensitivity of the exciton absorptions of the amide I band to the H-bond pattern between β-strands. Due to the ^13^C=O backbone isotopic substitution, the main β-sheet amide I signal splits with the frequencies and relative intensities of the two new (^12^C and ^13^C) bands reporting on the parallel or antiparallel orientation of the strands in the β-sheet. Aligned ^13^C labels (as in parallel sheets) lead to a new, shifted ^13^C absorption at a lower frequency below 1600 cm^–1^ with a weaker intensity compared to the main ^12^C absorption peak. Dispersed ^13^C labels in the peptide (as in antiparallel sheets) are more strongly coupled to ^12^C amides and lead to an observed frequency closer to the main ^12^C exciton peak, often well above 1600 cm^–1^ and with a stronger relative intensity than if the labels were aligned with each other. The high-frequency amide I band at around 1685 cm^–1^, when present, remains a single transition as the isotope substitution does not result in a splitting of this peak.^52,53^ We observe, however, that this signal does display a subtle shift with the introduction of single ^13^C labels due to the delocalized nature of the high-frequency absorption (see Figure S13).

**Figure 3:**
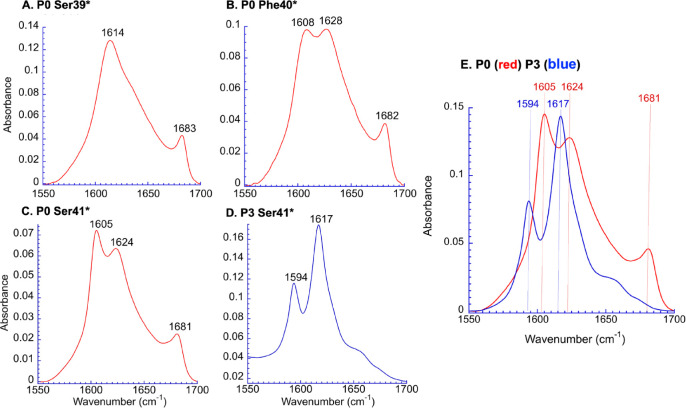
(A–D) IE FTIR spectra after 24 h of incubation at ambient temperatures of P0 and P3 with an intrinsic vibrational probe, ^13^C=O, placed in the backbone amide at the residue indicated with an asterisk (Figure 1C). (E) An overlay of P0 Ser41* and P3 Ser41* (normalized to the strongest intensity band).

The P0 IE FTIR spectral data for Gly38*, Ser39*, and Val48* (Figures 3A and S14), obtained at 2.0 mM peptide in water at pD 8.0, look relatively similar to those of unlabeled P0 (Figure 2), with a single low-frequency amide carbonyl signal. Difference amide I spectra vs the unlabeled peptide (Figure S13), however, indicate that these labeled amide residues are still involved in the β-sheet but are coupled to fewer ^12^C amides, suggesting that they are at the periphery of the β-sheet. For P0 with Phe40* and Ser41* (Figure 3B,C), the fully symmetric exciton peak is split into two similarly strong and discrete bands at 1605–1610 cm^–1^ (for ^13^C=O strongly coupled to many ^12^C=O) and 1620–1630 cm^–1^ (for a mode mainly containing remaining ^12^C=O). This spectral splitting pattern implies an antiparallel secondary structure, which is also consistent with the presence of the high-frequency amide exciton band at around 1685 cm^–1^. In contrast, the spectrum of P3 labeled with ^13^C-labeled Ser41* lacks the 1685 cm^–1^ band and displays a considerably weaker, sharp band at 1594 cm^–1^, while the main symmetric exciton band, mainly from ^12^C=O amides, remains at 1617 cm^–1^ (Figure 3D). For P3 Ser41*, the pronounced frequency shift of the new band introduced by the ^13^C label to below 1600 cm^–1^ with a relative intensity weaker than that seen in P0 with F40* and Ser41* substitutions is a clear indicator of a parallel strand arrangement.

#### 2.1.3 Strand Reorientation for P0–P2

**Figure 4:**
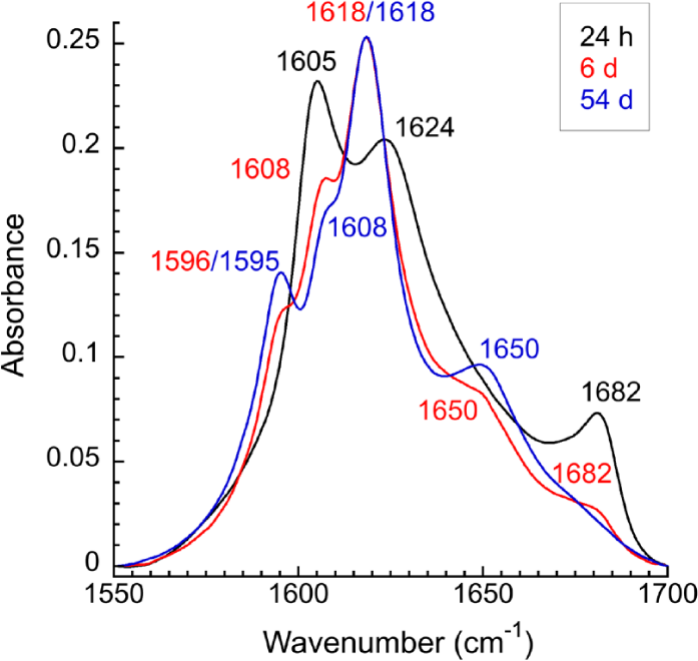
IE FTIR spectral data showing the conversion over time of P0 Ser41* from an antiparallel to a predominantly parallel β-sheet arrangement at 2.0 mM in water and pD 8.0, with time points taken at 24 h (black), 6 days (red), and 54 days (blue).

In the IE FTIR experiments of P0 Ser41*, distinct changes in the amide I region were observed over time. A spectrum of P0 taken after 24 h shows the signature bands of the antiparallel strand arrangement at 1605, 1624, and 1682 cm^–1^ (Figure 4). These peaks decrease in intensity over 6 days in favor of new signals appearing at 1595 and 1618 cm^–1^. This time dependence supports a reorganization of the β-sheet from an antiparallel to parallel strand orientation. The disappearance of the high-frequency peak at 1682 cm^–1^ provides further evidence for a secondary structural change. Concurrently with these spectral changes, the initially clear gel becomes cloudy with a visible white precipitate (Figure S15). The signal centered at around 1650 cm^–1^ is slightly more prominent on day 54 (Figure 4), suggesting a small amount of random coil in the sample, potentially due to marginal peptide degradation after more than 7 weeks in solution. Overall, the FTIR spectra show that the day 54 sample is still overwhelmingly β sheet in structure. No degradation of P0 was observed over two weeks in solution as assessed by mass spectrometry analysis (Figure S16), covering the duration of the majority of the experiments conducted in this study.

The time-dependent secondary structure reorganization of P0 from a kinetically favored antiparallel configuration to a thermodynamically favored parallel structure exhibits high sensitivity to minor differences in sample concentration and various factors such as sample container surface material (glass versus plastic and type of plastic), ionic strength, and identity of the peptides’ counterions. These variables collectively contribute to the complex behavior of P0, which usually yields a clear gel upon preparation but occasionally turns cloudy (Figure S15) with different apparent viscosities (as determined by visual inspection and rheology). The cloudiness coincides with the appearance of the IR signals corresponding to a parallel arrangement, which may indicate that the antiparallel fibrils are more colloidally stable than those composed of parallel β-sheets. While the self-assembly kinetics can be surface-catalyzed through heterogeneous primary nucleation, the final equilibrium state is determined by thermodynamics and is independent of the kinetic pathway.^54,55^

P1–P3 were similarly prepared with ^13^C=O-labeled Ser41*. The time-dependent spectra for P3 all support the presence of parallel β-sheets (Figure S17), consistent with previous FTIR studies of the unlabeled P3 peptide with no signal at 1685 cm^–1^ (Figure S5). For P2 Ser41* at both 1.0 and 2.0 mM (pD 8.0), parallel β-sheets are observed from day 1 and beyond (Figure S18). These IE FTIR data are consistent with the time-dependent IR spectra obtained for unlabeled P2, which show a time-dependent decrease in the intensity of the high-frequency band of ca. 1685 cm^–1^ at pD 7.0 and 8.0 (Figure S4). The IE FTIR data for labeled P1 is less clear-cut, as both parallel and antiparallel β-sheets are present initially. The parallel signal, however, disappears over time to leave a mixture of antiparallel sheets and a random coil, as indicated by the broad signal around 1650 cm^–1^ (Figure S19). At high pH (9–10) and more slowly at pH 7–8, a reorganization from antiparallel to parallel is seen for unlabeled P1 (Figure S3). Taken together, these data suggest that the strand orientation for P1 is highly sensitive to minor changes in sample preparation and that the parallel and antiparallel strand arrangements are similar in free energy.

**Figure 5:**
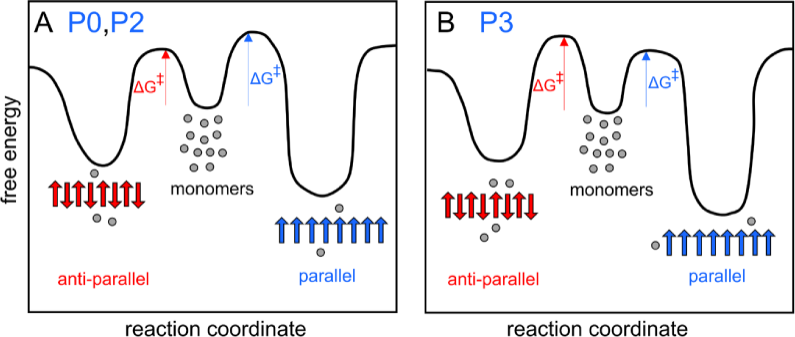
An energy landscape schematic for (A) P0 and P2, where the primary nucleation energy barrier is lower for an antiparallel than a parallel arrangement but where parallel is thermodynamically more stable, and (B) P3, where the barrier is lower for a parallel than an antiparallel arrangement and where parallel is also the thermodynamic product. While in this schematic, the conversion from one structure to the other is shown to go via aggregate dissociation to monomers, an alternative pathway for strand rearrangement could be direct conversion.

Figure 5A illustrates the time-dependent changes for P0 and P2, which undergo a transition from antiparallel to parallel arrangements. These changes, inferred from the FTIR spectra, imply that the barrier for primary nucleation is lower for one aggregated state than the other and that the states are separated by a high energy barrier. This would yield first a kinetically favored state that is then slowly converted to a state of lower free energy. In contrast, P3 forms only parallel sheets, indicating that its nucleation barrier to the most stable state is the lowest (Figure 5B). This behavior differs from cases like α-synuclein, where nearly equal barriers for the primary nucleation of two similar aggregated states result in similar initial populations across samples. Over time, these samples converge to the more stable fibril morphology, suggesting a high energy barrier between the states.^56^

Calculations using tabulated data for residue–residue interactions^57^ imply that there may be only a small difference in thermodynamic stability between the antiparallel and parallel arrangements. For most peptides, the free energy difference for the formation of a dimer is only within 1 kT; in other words, negligible (Figure S20). A parallel dimer arrangement appears to be favored over an antiparallel dimer for P1, P3, and P5. The packing into higher-order oligomers and fibrils may further modulate the stability differences. P0–P9 were also subjected to structural predictions using AlphaFold2^58^ to test whether this software could reproduce or help rationalize the observed behavior. We asked how many chains are needed in the query for the prediction to be dominated by a parallel arrangement. P1 and P3 stood out as requiring fewer chains for the parallel arrangement compared with the general trend (Figure S21), suggesting that they may be more prone to forming fibrils with parallel peptides. Across the hydrogel peptide series, manual calculations and AlphaFold2 predictions best reproduced the stable behavior of P3 and the more condition-sensitive behavior of P0. These models predict, however, that P1 should favor a parallel strand orientation, while our data show that the free energy for antiparallel and parallel arrangements is likely quite similar. This discrepancy could be explained if P1 is prone to forming alternate-register β-sheets with overhanging residues, which is not accounted for in these other models. When comparing the solvent-mediated free energies of alternate potential parallel and antiparallel β-sheet registers for P0–P10, P1 is the only peptide for which the one-residue out-of-register antiparallel and in-register parallel dimers have similar stabilities (Figure S22).

#### 2.1.4 Probing the β-Sheet Core in P0 Fibrils

The IE FTIR studies of ^13^C=O-labeled P0 variants imply that the *N-* and C-terminal ends of the β-strands of the fibril structure are frayed, which led us to the question how many residues are part of the β-sheet core? The nitrile functional group, C≡N, is one of the smallest extrinsic vibrational probes available. Carbon-bound nitriles contain a stretching mode in the IR at around 2220–2250 cm^–1^, which for peptides and proteins lies in a typically unpopulated region. In an aqueous environment, the position of the nitrile stretch in the IR is highly sensitive to the degree of H-bonding to surrounding water molecules and can therefore be used to probe the local environment of a residue within the fibrillar superstructure.^59,60^

**Figure 6:**
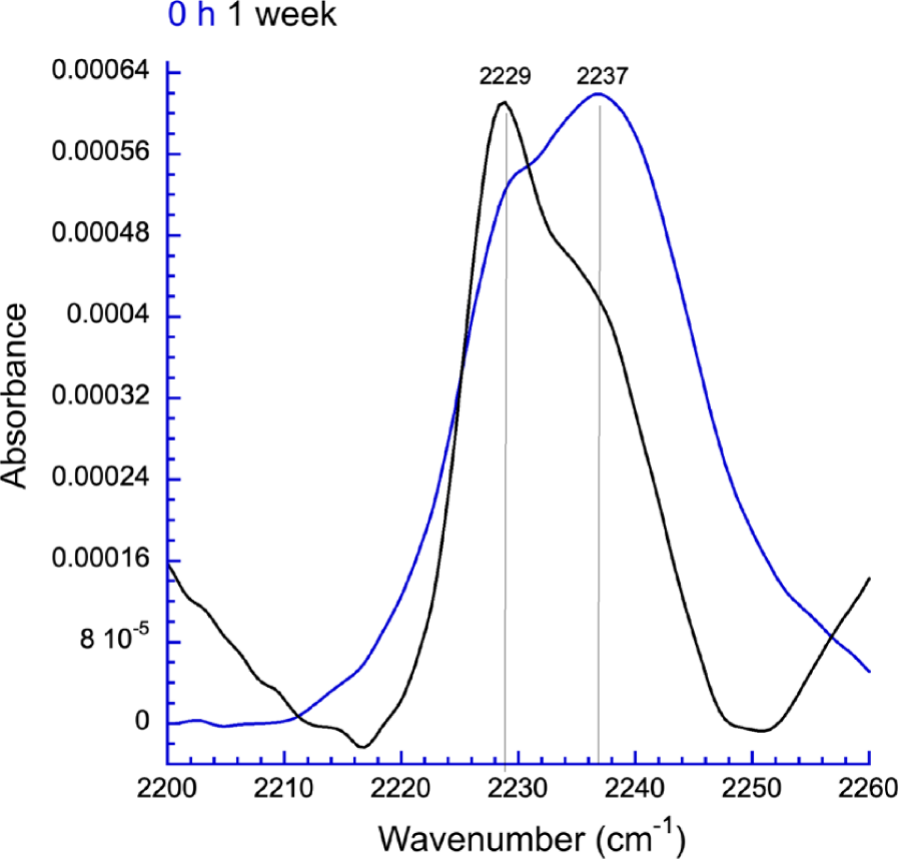
A sample of F40F_CN_ (1.0 mM in H_2_O at pH 8.0) was incubated at ambient temperature and analyzed immediately (blue, predominantly water-exposed) and after 1 week (black, predominantly fibril-embedded).

Three mutants of P0 were made, incorporating the unnatural amino acid 4-cyano-phenylalanine, Phe^CN^, in place of the aromatic residues Phe (denoted F40F_CN_) or Tyr (Y44F_CN_ and Y46F_CN_, Figure 1C), and time-dependent IR spectra were obtained. For F40F_CN_ at 1.0 mM (Figure 6), the nitrile region of the spectrum shows two partly overlapping bands, centered at 2229 and 2237 cm^–1^ (Figure S23 and Table S1). The higher frequency band at 2237 cm^–1^ is indicative of a nitrile fully exposed and H-bonding with water. The lower frequency band at 2229 at cm^–1^ is close to that reported for a Phe^CN^ nitrile in a non-H-bonding embedded environment (2228 cm^–1^).^59^ A transition between the two populations, from water-exposed to more fibril-embedded, is clearly seen over time. The amide region of the spectrum shows signals at ca. 1620 and 1685 cm^–1^, confirming that this vibrational probe does not perturb the formation of the expected β-sheet (Figure S24). The data for Y44F_CN_ and Y46F_CN_ at 1.0 mM (Figures S25–S28 and Tables S2 and S3) are consistent with these residues being more deeply embedded in the fibril structure than F40F_CN_.^60^

The combined IR data for the Phe^CN^ mutants and the ^13^C=O-labeled Gly38*, Ser39*, and Val48* variants, for which the amide carbonyl signal around 1620 cm^–1^ is not fully split, are consistent with the core of the fibril structure spanning the central 7 residues, from Phe40 to Tyr46. These 7 central residues are amide–amide-H-bonded to the adjacent peptide strands within the same sheet, and the Phe40, Tyr44, and Tyr46 residues are involved in sheet–sheet hydrophobic interactions along a defined hydrophobic “face” of the fibrils that encompass residues 40, 42, 44, and 46. The 7 central residues, Phe40 to Tyr46, support the fibril stability and the peptide self-assembly into organized structures. The arrangement and chemical properties of residues Phe40 to Tyr46 enable them to mediate both intrasheet and intersheet interactions, crucial for forming stable fibrillar structures.

### 2.2 Macromolecular Superstructure

#### 2.2.1 Cryo-TEM: Fibril and Network Morphology of P0–P7

**Figure 7:**
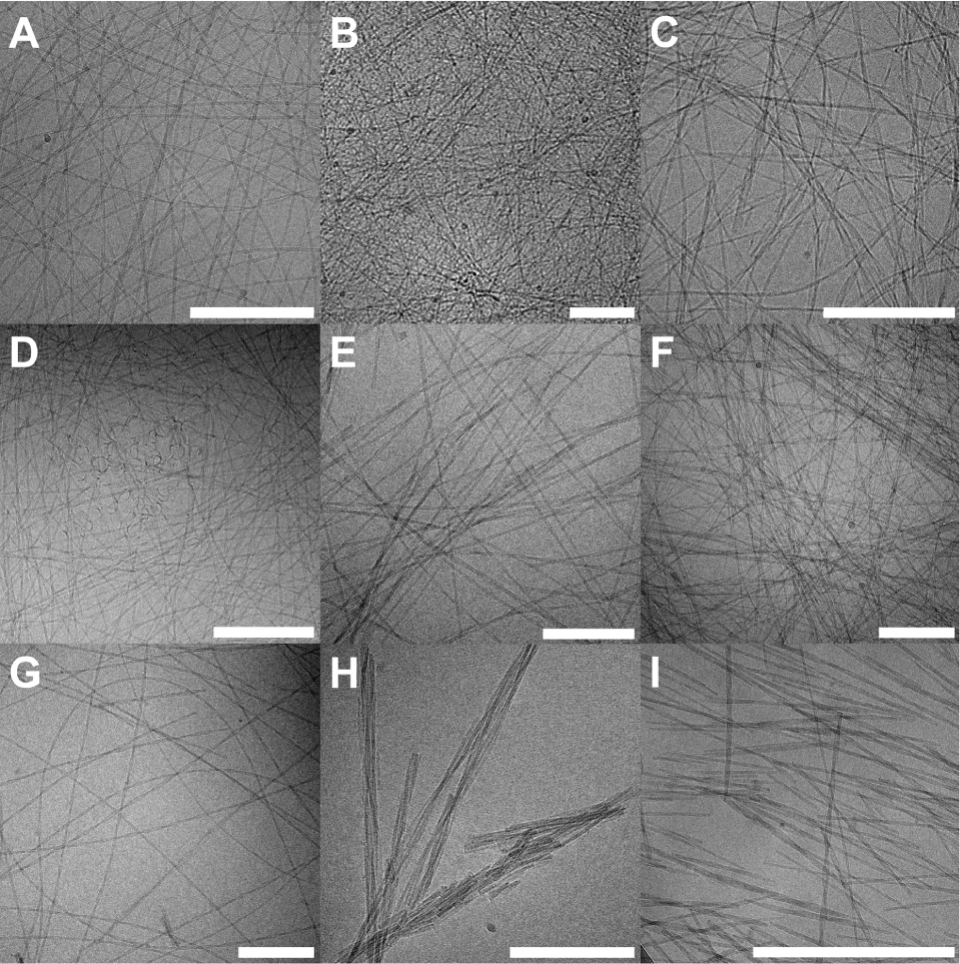
Cryo-TEM images of P0–P6 (for additional images, see Figure S29). (A) P0, (B) P1, (C) P2, (D) P3, (E) P4, (F) P5, and (G) P6. Small curved objects are observed for P3 (D), which are attributed to an early intermediate in fibril formation. Shorter fibrils are more frequently observed for sequences P5 (H) and P6 (I). The images were taken after 1 week of incubation at 21 °C in pH 8.0 water and at a concentration of 2.0 mM except for panels B and C, which were imaged from samples prepared at 100 μM. The white scale bar in all images represents 200 nm.

Cryo*-*TEM imaging was used to assess the presence of fibrils for peptides P0–P7 at pH 8.0 (Figures 7, S29, and S30). The images confirm that under these conditions, peptides P0–P6 all form thin, long fibrils. In a comparison of P0–P6, despite the loss of several amino acid residues, the TEM images exhibit similar fibrillar structures. By visual inspection, however, it is apparent that P4–P6 separate into two phases after hydrogel preparation, indicative of colloidal instability (see images in Figures S6–S8), while P0–P3 form homogeneous, clear hydrogels (Figures S2–S5). Cryo*-*TEM images also show that P7, at its solubility limit in water (1.0 mM, pH 8.0), contains no fibrils (Figure S30A). The absence of fibrils is consistent with the lack of β-sheet content in the FTIR spectra and the inability of P7 to form a hydrogel under these conditions. Cryo-TEM images were not obtained for the shorter peptides P8–P10, which, similar to P7, did not generate hydrogels nor show any indication of structured materials present, as assessed by amide I FTIR.

The fibrils observed in the cryo*-*TEM samples are typically on the micrometer length scale and extend beyond the image window. Shorter fibrils are also often observed with P4–P6 (Figures 7H,I, S29H,I), suggesting that they are more likely to naturally form shorter fibrils than P0–P3 or that they more easily fragment during cryo*-*TEM sample preparation. In the case of P3, small, curly objects are also present in clusters throughout the samples (Figure 7D) and are particularly seen in images taken at early time points.

#### 2.2.2 SAXS/WAXS: Fibril and Network Morphology of P0–P7

SAXS and WAXS measurements were used to complement the cryo-TEM images and to obtain a more complete understanding of the fibrillar structure of the samples. The SAXS region of the scattering curve, generally considered to be below *q* < 0.5 Å^–1^, provides nanometer-scale shape characteristics, whereas the WAXS region, *q* > 0.5 Å^–1^, yields higher, atomic (Ångström) scale information.

**Figure 8:**
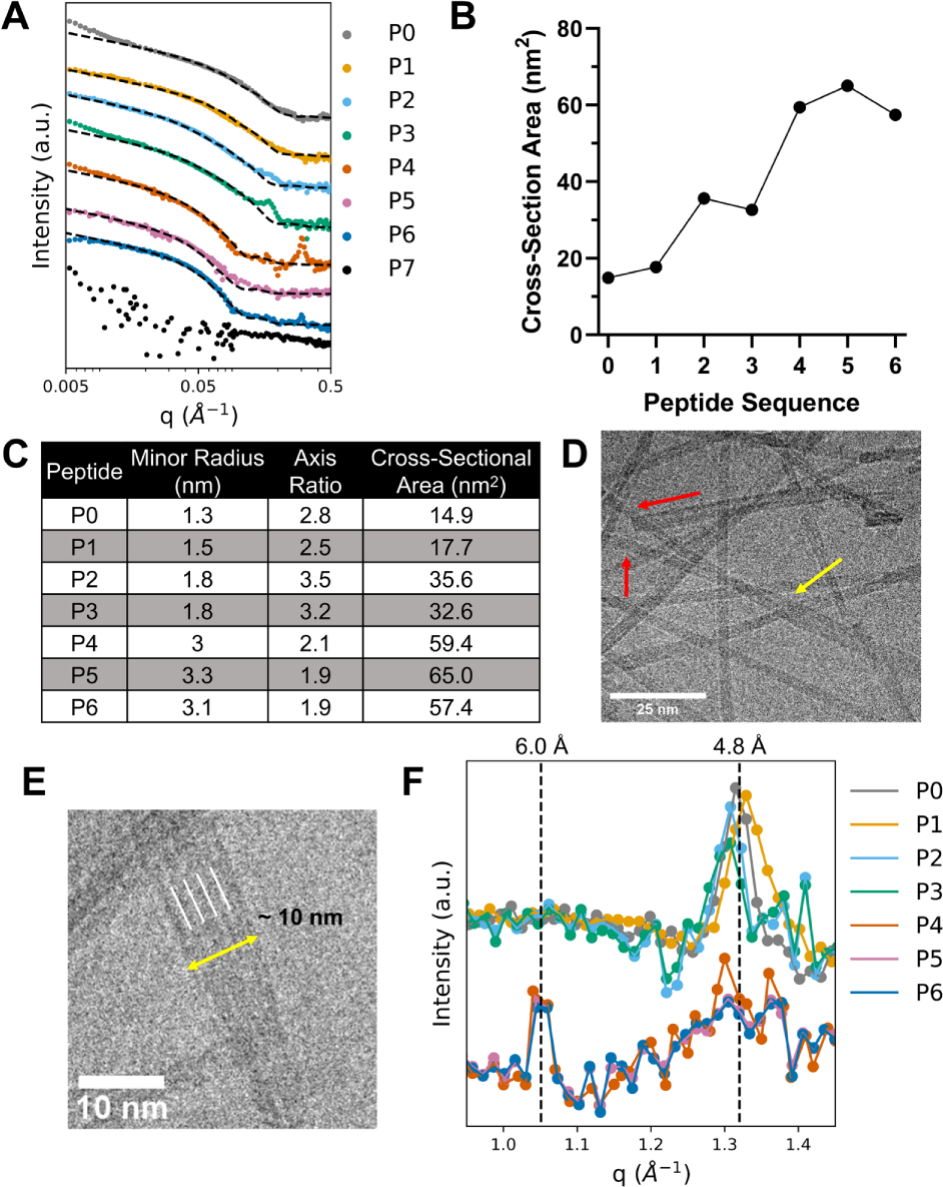
(A) SAXS curves (0.004 < *q* < 0.5 Å^–1^) of 2.0 mM P0–P6 and 1.0 mM P7 after 7 days of incubation at 21 °C and pH 8.0 with elliptical cylinder model fits shown as black dashed lines. (B) The relationship between the cross-section area and the sequence demonstrates a steady increase in the thickness of the fibrils as residues are removed from the *N-*terminus. (C) Table of the fibril dimensions for each peptide at day 7 as determined by fitting each scattering curve to an elliptical cylinder model. (D) Cryo*-*TEM image of P4 at 100k magnification after 7 days of incubation at 21 °C showing two smaller fibrils (red arrows) twisting together into a bundle (yellow arrow). (E) Close-up P5 cryo-TEM image (after 7 days of incubation at 21 °C) of a fibril bundle composed of approximately 5 fibrils and a diameter of ∼10 nm (yellow line). White lines show boundaries between fibrils in the cluster, as approximated by ImageJ pixel intensity analysis. (F) Baseline-subtracted and area-normalized plot of the WAXS region of the scattering curve, where P0–P3 and P4–P6 are offset from each other, show a peak at 5.9 Å (1.05 Å^–1^) present in P4–P6 and a peak at 4.8 Å (1.32 Å^–1^) present in P0–P3.

The SAXS data (Figure 8A) confirm the formation of fibrils for P0–P6. For P7, no significant scattering is observed, consistent with the lack of peptide self-assembly and gel formation. To extract information about the average fibril dimensions for P0–P6, an elliptical cylinder scattering model was fitted to the data (dashed lines in Figure 8A). Fitting the data provided physical parameters for the fibrils, including the minor semiaxis, the axis ratio, and the corresponding cross-sectional fibril area (Figure 8B,C and Table S4). As illustrated in Figure 8B, the fibril cross-sectional area generally increases from P0 to P6 as the peptides become shorter, and is particularly apparent in the transition from P3 to P4. Additionally, the shape of the scattering curves for P4–P6 deviates from the longer sequences in the midq regime around 0.08 Å^–1^ (Figures 8A and S31). The fits for P0–P3 display cross-sectional areas below 40 nm^2^, whereas those for P4–P6 are between 55 and 65 nm^2^ (Figures 8B and 8C). Taken together, these data suggest that a shortening of the peptide sequence increases fibril–fibril attractive interactions, leading to the formation of larger fibril aggregates, which is consistent with the fibril bundling observed for P4–P6 in the cryo-TEM images (Figures 7H,I, 8D,E, S29H,I, and S30B). This bundling likely contributes to the propensity for these sequences to phase-separate.

The WAXS region signals (Figure 8F) show a peak at *q* = 1.3 Å^–1^ for sequences P0–P3, corresponding to a periodic repeat distance of 4.8 Å that we associate with β-strand separation. This reflection is often observed in amyloid fibrils such as those generated by amyloid β42 or α-synuclein.^24,61^ In P4–P6, there is instead a peak at *q* = 1.05 Å^–1^, corresponding to a periodic repeat distance of 6.0 Å, indicating a slightly different packing of the peptides within the fibrils. Most likely, the β sheets here still have a 4.8 Å periodic β-strand repeat distance in the fibril direction. The 6.0 Å reflection could thus be a characteristic distance in the plane perpendicular to the fibril-strand direction, such as the periodic lamination distance between layered β-sheets. This phenomenon has been previously observed in the short synthetic peptides A_8_K and A_10_K. There, due to an oblique 2D packing of the peptide molecules, the β-sheet is not normal to any crystallographic axis, and a 4.8 Å reflection is therefore not observed; however, a 5.5 Å peak corresponding to the lamination distance was clearly observed.^62,63^

In P5 after 1 day (Figure S32) and P4 and P6 after 7 days (Figure 8A) of incubation at 21 °C, there is also a sharp peak present at *q* = 0.305 Å^–1^ (2.1 nm). This is likely caused by the lateral assembly of multiple fibrils to form the large, layered sheet-like structures observed in cryo-TEM (Figures 8E and S30B). For example, images of P5 contain bundles of multiple fibrils lining up next to each other to form larger fibrils with an estimated width of about 2 nm (Figure 8E). Similar lateral assemblies were reported for NACore (H_2_N–GAVVTGVTAVA–OH) fibers that have a 3D crystalline order.^64^ A crystal ordering in three dimensions would indicate that the fibril formation is a precipitation of a crystalline peptide phase and would thus be consistent with the macroscopic phase separation observed visually for P4–P6. Interestingly, both P2 and P3 also exhibit a broad peak at *q* = 0.172 Å^–1^, which corresponds to a repeated dimension of 3.7 nm (Figure 8A). Although the exact cause of this peak is unknown, it closely corresponds to the expected diameter of the fibrils and may be a consequence of multiple fibrils bundling together into sheets. This bundling phenomenon, however, was not captured by cryo-TEM.

### 2.3 Material Properties

#### 2.3.1 Rheological Characterization of P2 and P3 Hydrogels

A key property of hydrogels is their viscoelastic behavior and ability to resist deformation. Small oscillatory rheological measurements are often used to characterize these properties through the storage (*G*′) and loss (*G*″) moduli of gels. Using oscillatory experiments, it is possible to uncouple the elastic solid-like character of a material and its ability to store energy (*G*′) from the dissipation of energy from the material (*G*”). The magnitude of G′ correlates to gel stiffness.^65^ The hydrogel-forming peptides P2 and P3 were investigated by rheology to attempt to relate the fibril morphology to hydrogel mechanical properties. These two sequences were selected because they had the most consistent hydrogel-forming properties of the 11 peptides investigated.

**Figure 9:**
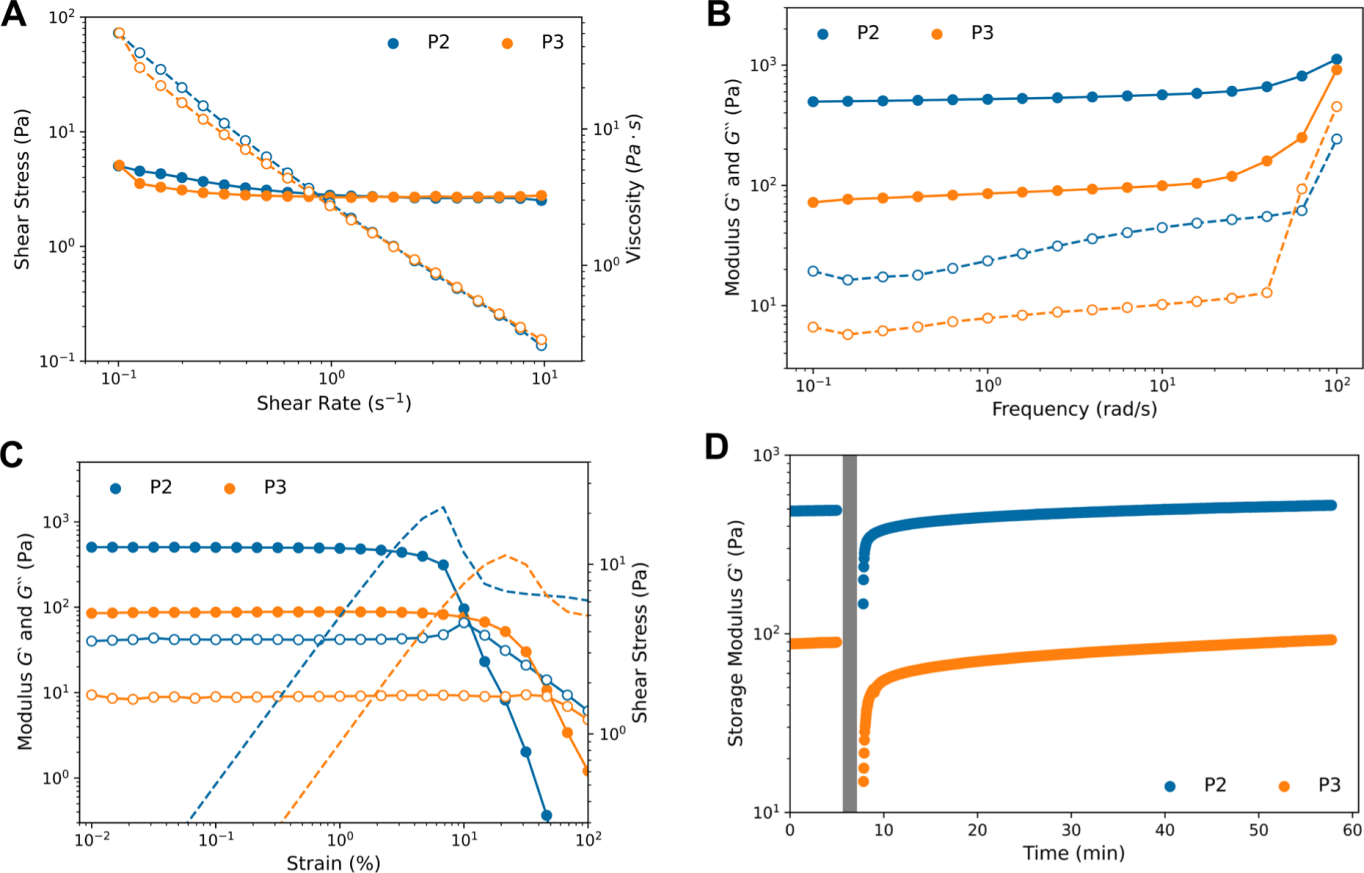
Rheological measurements on P2 and P3 hydrogels after incubation at ambient temperature for 1 week. (A) Flow curve of P2 and P3 with shear stress (filled circles) and viscosity (open circles) plotted against shear rate (0–10 s^–1^). (B) Frequency sweeps of *G*′ (filled circles) and *G*″ (open circles) with a frequency range of 0.1–100 rad/s. (C) Amplitude sweeps plotting *G*′ (filled circles), *G*″ (open circles), and shear stress (dotted line) over strain values of 0.01–100%. (D) The recovery time of *G*′ at a strain of 0.1% was measured after deformation induced by a flow curve experiment. The flow curve was conducted at a shear rate ranging from 0.01 to 300 s^–1^, which corresponds to a logarithmic strain ramp of 37.6–695000% over 170 s (gray area).

Flow curve data for P2 and P3 demonstrate their shear thinning behavior as the viscosity decreases with an increasing shear rate with a slope of ca. −1.1 (Figure 9A). Zero-shear viscosity is not observed in the investigated shear rate range, indicating that P2 and P3 are yielding materials in which the hydrogels can recover back to their original state over the tested shear rates. Frequency-sweep experiments confirm that P2 and P3 are stable hydrogels in which the G′and G″ moduli are frequency-independent up to 20 rad/s (Figure 9B). Amplitude sweep experiments demonstrate that P2 and P3 are linearly viscoelastic up to 2% and 10% strain, respectively, as seen by the stability of G′ in these ranges (Figure 9C). P2 and P3 lose gel character and exhibit liquid-like flow at strains above 10% and 60% strain, respectively, as indicated by *G*″ > *G*′ (Figure 9C). After this strain-induced loss of gel character, P2 recovers to ca. 75% and P3 to ca. 60% of their initial *G*′ values within 2 min, and a complete recovery to the initial *G*′ value takes place after 30 and 45 min for P2 and P3, respectively (Figure 9).

#### 2.3.2 Longitudinal Characterization of P2 and P3 Gels

**Figure 10:**
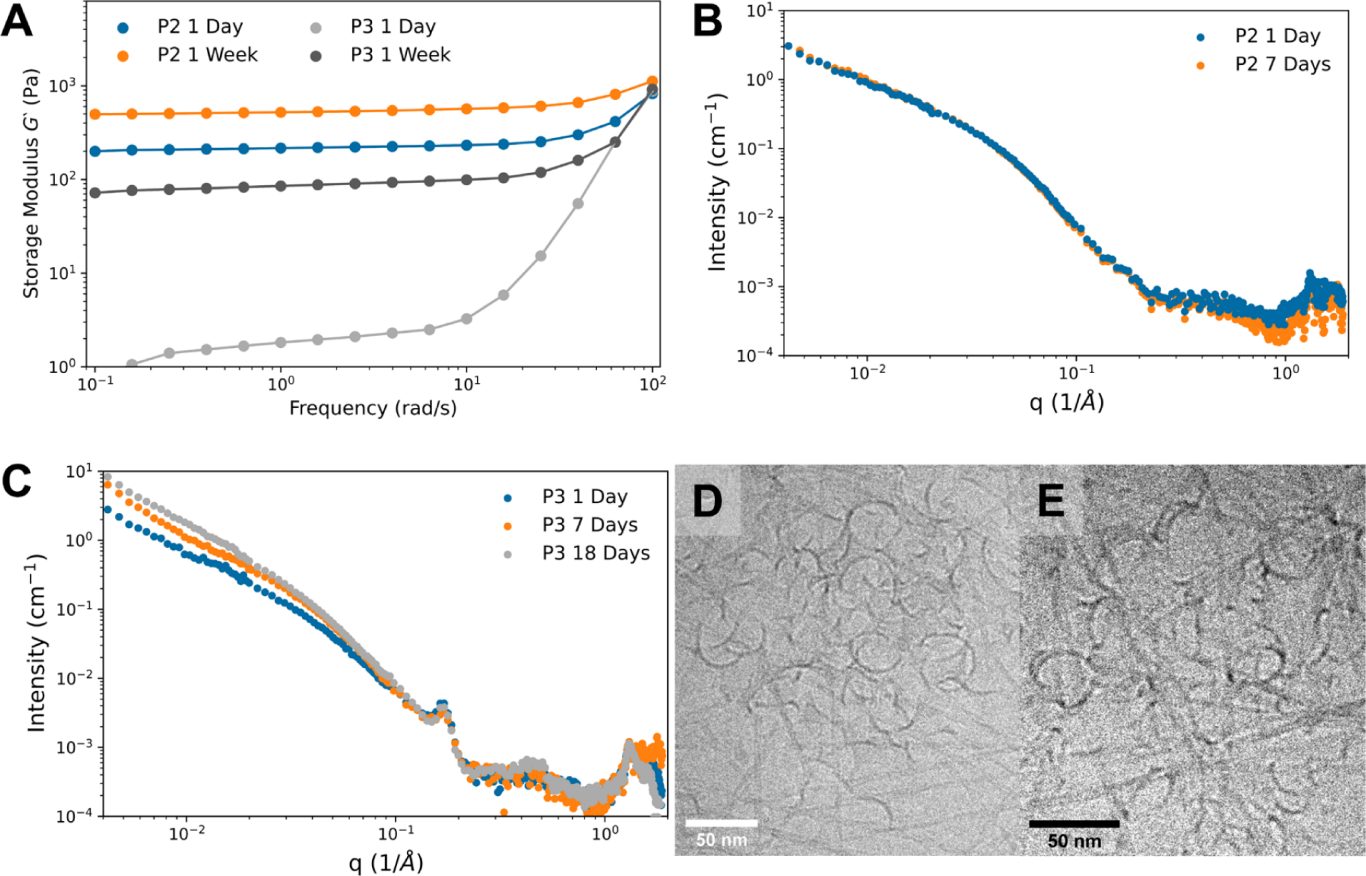
(A) The storage modulus (*G*’) plotted against angular frequency after the 2.0 mM pH 8.0 samples of P2 and P3 were incubated for 24 h and 1 week at 21 °C. The storage modulus (*G*’) for both peptides increases over the course of a week. (B, C) Scattering curves of P2 (B) and P3 (C) after various incubation periods at 21 °C. P2 scattering curves do not change, suggesting that the fibril morphology has reached a steady state while P3 continues to change over 18 days of incubation. (D,E) Cryo-TEM images of curly objects present in P3 after 3 h (D) and 7 days (E) of incubation at 21 °C showing that the curled structures persist over the 1-week incubation period. The scale bars represent 50 nm.

The time evolution of hydrogelation was explored by conducting rheological measurements of P2 and P3 hydrogels 1 and 7 days after sample preparation. Over the course of one week, the storage modulus of P2 modestly increases and P3 significantly increases, suggesting that the materials are not at equilibrium (Figure 10A). Thus, longitudinal X-ray scattering measurements of P2 and P3 fibrils were performed at the same peptide concentrations as the rheology studies to elucidate the cause of this increase in the hydrogel strength over time.

SAXS curves of P2 at 1 and 7 days overlap almost completely, indicating no changes in the form factor (i.e., fibril morphology) or fibril concentration over time (Figure 10B). This suggests that the increase in strength of the P2 hydrogel between 1 and 7 days of incubation may be related to the changes in the peptide secondary structure that can occur over this period, as observed by FTIR (Figure S4). In contrast, over a week of incubation, the low *q*-region of the P3 SAXS curve changes for P3 (Figure 10C). To determine whether P3 had reached a steady state within 7 days, we conducted an additional SAXS experiment after 18 days of incubation. The curve only changed slightly from the 7-day curve, suggesting that a steady state fibril morphology is mostly achieved after 7 days of incubation. Cryo*-*TEM analysis of P3 revealed the presence of low-aspect-ratio “curved” polymorphs. These “curved” structures appear in images taken after both 3 h and 7 days of incubation of P3 (Figure 10D,E) and are not observed at any time point for P2. Therefore, one explanation for the observed changes in the P3 SAXS curves and rheology over time may be due to the conversion of these polymorphs into extended fibrils.

#### 2.3.3 Peptide Length Dependence of Self-Assembly and Gelation

A main goal of this study was to investigate a naturally occurring peptide, P0, derived from semenogelin I, through a progressive stepwise amino acid truncation to identify the minimal gel-forming portion within this sequence. Three groups emerge. The longest peptides, P0–P3 (group 1), form clear hydrogels, as determined by visual inspection or rheology. P4–P6 (group 2) are colloidally unstable and phase-separate. The shortest peptides, P7–P10 (group 3), lack a secondary structure and the ability to form a gel. While cryo-TEM images show that group 2 peptides form long μm-sized fibrils similar in appearance to those of group 1, the group 2 aggregates appear more fragile with frequently observed shorter “broken” fibrils that are more prone to cluster. SAXS/WAXS data for group 1 peptides show a reflection at 4.8 Å, consistent with the β-sheet interstrand distance. This reflection is not readily apparent for group 2, where other reflections are seen at 2.1 Å (P5 and P6) and 6.0 Å (P4–P6). The amide IR spectra show that P4–P6 consist of parallel β-sheets; however, other sharp signals, in addition to 1620 cm^–1^, are clearly visible at 1640 and 1660 cm^–1^ and are particularly striking in the IR spectra of P4 (pD 5.3, Figure S6), P5 (pD 5–8, Figure S7), and P6 (pD 8.0, Figure S8). These features are indicative of the presence of well-organized but different structures from those generated by the group 1 peptides and might even be composed of more than one type of β-sheet. For the longer group 1 peptides, more surface area would be available for interactions with nearby strands, which might be expected to provide increased stability to the growing fibril as it assembles.^66,67^ In the case of P0, however, and by inference P1 and P2, the three *N-* terminal residues appear to engage in H-bonding between strands to a lesser extent than the core residues Phe40 through Tyr46, as determined by IE FTIR. These three *N-*terminal residues may thereby contribute to the relative instability of the P0, P1, and P2 sheet structures, which are prone to reorganization.

#### 2.3.4 Amino Acid Determinants in Supramolecular and Macromolecular Assembly

Although the truncated series of peptides in this study have different lengths, we do not observe a continuous change in the properties with the sequential removal of one amino acid residue at a time. Instead, our investigations have identified three amino acid residues that appear to be of particular importance, namely, Ser39, Phe40, and Gln43. Removal of any of these residues, as the sequence shortens, leads to major changes in properties, as manifested in a change in morphology and hydrogelation properties. In going from P2 to P3, with the loss of Ser39, the orientation of the strands within the β-sheet is no longer prone to rearrangement but remains stably parallel. While P2 and P3 only differ by one residue and appear similar in SAXS, WAXS, and cryo-TEM, the *G*′ of P2 is close to an order of magnitude higher than P3 after 1 week of incubation. Thus, losing serine does not appear to significantly change the fibril morphology but greatly impacts the rheological properties of the hydrogel. One possible reason for the difference in rheological properties could be the different strand orientations in the fibrils (antiparallel for P2, with a slow conversion to parallel and parallel for P3). Similarly, amyloid α-synuclein fibers, in response to changes in pH^24^ and temperature,^23^ have been reported to undergo molecular rearrangements without impacting the observed SAXS curves. The difference in *G*′ between P2 and P3 is accompanied by the presence of the observed curly objects in P3, which may be an early aggregation intermediate whose presence might weaken the gel.

In peptides P0–P3, there are three aromatic residues present, Phe40, Tyr44, and Tyr46. The loss of Phe40 on going from P3 to P4 results in a loss of fibril colloidal stability and IR spectral differences. New WAXS reflections are observed in P4 samples at 2.1 and 6.0 Å, indicative of the presence of a new highly ordered nanostructure distinct from that of the longer sequences. Phenylalanine is prone to π–π stacking and is known to play an important role in peptide self-assembly. For example, the dimer Fmoc–Phe–Phe self-assembles into various supramolecular structures and can also form a hydrogel.^32,68^ In the case of P3, the *N-*terminal Phe residue might interact favorably with the Phe residues on adjacent strands in the parallel β-sheet. Phe40 might serve as an important stabilizing residue for the sheet structure, where its loss results in a change in packing between sheets that leads to a decreased colloidal stability of the hydrogel network. For P4 and shorter peptides, an increased presence of shorter fibrils and fibril clusters is also observed in cryo-TEM images (Figures 7H,I and S30B). Losing this residue may also disrupt the delicate balance between stabilizing attractive hydrophobic forces and repulsive electrostatic interactions, which are required for a self-supporting hydrogel network of long fibrils.

The removal of Gln43 from P6 to yield P7 results in a loss of fibril formation. Many studies have demonstrated the importance of glutamine in the formation of amyloid-like fibrils, especially in Gln-rich sequences.^69^ In P0, glutamine is the central residue in the 13mer sequence, which may be able to stabilize inter-β-sheet interactions regardless of the relative orientation of the strands. In the cases of P3–P6, which all form only parallel sheets, Gln could provide important stabilizing interactions that favor one orientation over another. The presence of Gln might contribute to the thermodynamic stability of the parallel arrangement for P0, P2, and P3. Electrostatic repulsion between the charged C-terminal Asp residues on adjacent strands may also contribute to the initial formation of antiparallel β-sheets and prevent assembly altogether in the short sequences P7–P10.

## 3 Conclusion

Our comparative structural and material properties investigations have led to the identification of P2 and P3 as promising biomaterials for further investigation. Previous microfluidics experiments have shown that P0 can form microgels suitable for drug encapsulation.^38,39^ Here, we show that P2 and P3 display similar properties to peptide hydrogels currently being investigated for biomedical applications.^70,71^ Compared to synthetic hydrogel-forming peptide sequences, these peptides are derived from a human protein and, therefore, may be less immunogenic, further improving their potential as biomaterials for drug delivery for chronic illnesses and tissue engineering.

## 4 Experimental Materials and Methods

### 4.1 Materials

All solvents and reagents were purchased from commercial suppliers and used without further purification, unless otherwise indicated. *O*-(Benzotriazol-1-yl)-*N,N,N’N’*-tetramethyluronium hexafluorophosphate (HBTU), 1-hydroxybenzotriazole hydrate (HOBt hydrate), and Fmoc-protected amino acids were purchased from Advanced Chem Tech. Fmoc-4*-*cyano-phenylalanine was obtained from Sigma-Aldrich, and Amide Rink resin was purchased from ChemPrep. Sodium deuteroxide (NaOD) (30% w/v, 99.5% D), deuterium oxide (D_2_O) (99.9% D), and all ^13^C-labeled and Fmoc-protected amino acids used in this study were acquired from Cambridge Isotope Laboratories.

### 4.2 Methods

#### 4.2.1 Peptide Synthesis

All peptides were made employing a standard Fmoc (*N*-(9-fluorenyl)methoxycarbonyl) solid phase peptide synthesis protocol employing an Applied Biosystems 433A or a Gyros Protein Technologies Tribute Peptide Synthesizer. To minimize the formation of deletion mutants, each amino acid was added twice, using a double coupling procedure, to the growing chain with each coupling step using 0.5 mmol (5 molar eqv) of amino acid (0.3 mmol and 3 molar equiv in the case of Fmoc-4*-*cyano-phenylalanine and 0.25 mmol and 2.5 molar equiv for ^13^C-labeled amino acids) in a 1:1 mixture with HBTU. All peptides were made with capped ends using a RINK resin (0.10 mmol, 0.43 mmol/g) to furnish a *C-*terminal carboxamide and acetic anhydride in the final *N-*acetylation step. Fmoc deprotection was achieved with 20% piperidine in DMF.

The peptide was cleaved from the resin with TFA/thioanisole/1,2-ethanedithiol/anisole (v/v) 9.0:0.5:0.3:0.2 (5.0 mL) for 2–3 h at ambient temperature. The cleavage solution was then concentrated with a stream of N_2_ gas to a volume of approximately 1 mL, and the crude peptide was precipitated with the addition of ice-cold diethyl ether. The precipitated peptide was collected by filtration through a fine sintered glass funnel, redissolved in water and acetonitrile, and lyophilized.

Crude peptides were purified by reversed phase HPLC using a reversed phase Vydac C18 column (22 mm x 250 cm, 10–15 μm particle size, catalog number: 218TP101522), employing a Rainin Model SD-200 HPLC, equipped with a Dynamax Model UV-D Detector, and a linear gradient of doubly deionized H_2_O with 0.1% trifluoroacetic acid (solvent A) and CH_3_CN:H_2_O 9:1 with 0.1% trifluoroacetic acid (solvent B). The gradient used for S0 was 15–40% B over 25 min with the absorbance detected at 220 nm, and the remaining peptides were purified with either the same or a slightly modified linear gradient at 1% increase of B/min.

The identity and purity of all peptides were verified by electrospray or matrix-assisted laser desorption/ionization time-of-flight (MALDI-TOF) mass spectrometry (Bryn Mawr College, PA, at the Chemical Center, Lund University, Lund, Sweden, or at the Laboratory for Biological Mass Spectrometry at Texas A&M University, College Station, TX). Mass spectra were typically acquired using an Autoflex Speed MALDI TOF/TOF mass spectrometer (Bruker Daltonics) in positive reflector mode. A 1 μL peptide sample was mixed with 0.5 μL matrix solution, consisting of 5 mg/ml α-cyano-4-hydroxy cinnamic acid, 80% acetonitrile, and 0.1% TFA, and added to a MALDI stainless steel plate. All spectra were externally calibrated using Peptide Calibration Standard II (Bruker Daltonics) containing 9 internal standard peptides. The peptide of interest (P0 for Figure S16) was identified based on MSMS analysis and database search using Mascot and an in-house database containing the sequence of the P0 peptide.

#### 4.2.2 Hydrogel Sample Preparation

All samples were prepared in nonstick Eppendorf tubes (Axygen, Inc.). Prior to the final sample preparation (for all FTIR samples and when otherwise stated), the TFA counteranions present in the peptide sample after HPLC purification were exchanged for chloride using the following protocol: lyophilized peptide was dissolved in deionized D_2_O (1.0 mL). HCl was added at a molar ratio of 13.7:1 HCl:peptide. For the Phe^CN^ mutants, the sample was divided into two groups and lyophilized. One sample was then redissolved in H_2_O (to avoid obscuring the O–D stretches in the nitrile region) and the other in D_2_O (to avoid overlapping of the O-H bending vibrations in the amide I region) to a final concentration of 2.0 mM, unless otherwise specified. All other peptide samples were redissolved in D_2_O. The exchange of TFA^–^ to chloride may be verified by a lack of signal at 1672 cm^–1^, seen in the IR spectrum for the deuterium-exchanged TFA salt of P3, where the carbonyl signal from TFA is well separated from the amide I carbonyl high frequency stretch at 1684 cm^–1^ (Figure S1). On the day of the peptide sample preparation, D_2_O was placed in an Eppendorf tube, was then used as background when collecting data points (0 h, 48 h, 1 week, etc.), and was assumed to absorb the same amount of H_2_O over time as the peptide sample prepared on the same day. The concentration of the peptide solutions was verified with the UV–vis absorbance peak at 280 nm using a molar absorptivity of 2560 M^–1^ cm^–1^ for all sequences (P0–P7). The pH/pD of the samples was then corrected to 8.0 using small volumes of NaOH and HCl (or NaOD and DCl). After attaining the desired pH/pD, samples were incubated at ambient temperature (ca. 22 °C) and followed over 7 days (or as specified) to allow for hydrogelation. No degradation of P0 (as either the chloride or trifluoroacetate salt) was observed by MALDI-TOF/TOF MS over two weeks (Figure S16).

##### 4.2.2.1 IR Spectroscopy

FT IR was performed with a Bruker Vertex 70 spectrometer with a photovoltaic mercury cadmium telluride (MTC) detector. Before the spectral collection, the MCT detector was cooled with liquid nitrogen. A background spectrum (see hydrogel sample preparation above) was collected (6 μL of H_2_O or D_2_O), and the collection chamber was purged for 15 min before 6 μL of sample was loaded in between two CaF_2_ windows and separated by a 50-μm Teflon spacer in a 25-mm diameter demountable liquid cell (Harrick Scientific). After purging again, the IR spectrum was collected with 512 scans, with a resolution of 2 cm^–1^, from 900 cm^–1^ to 4000 cm^–1^ at time *t* = 0 h, 24 h, 48 h, and 1 week (and sometimes longer) after sample constitution, processed using OPUS spectroscopy software, and plotted with Origin or KaleidaGraph.

##### 4.2.2.2 Rheology

Rheology measurements were performed on an Anton Paar Physica MCR 301 instrument using a CP25-1 (diameter of 25 mm, angle of 1°) cone plate. A water trap was installed to prevent sample evaporation, and then, sample, 150–200 μL, was carefully plated on to the rheometer stage with a tipless 1 mL syringe to minimize gel deformation. Measurements were conducted with a 0.48 mm gap size at a temperature of 25 °C. All rheological measurements were done at a frequency, ω, of 10 rad/s with data collection every 2 s. Samples were left for 1 h at a steady strain sweep, γ, of 0.5% (within the linear viscoelastic regime for all sequences) for the gel samples to recover a steady state fibril network, as observed by a stabilization in the value of *G*′. Subsequently, frequency sweep (0.1–100 rad/s), strain sweep (γ = 0.01–100%), and flow curve (0.1–50 s^–1^) experiments were conducted. A time experiment in the linear viscoelastic regime (10 rad/s, 0.5%) for a 50 min period after the flow curve was used to estimate the recovery time of the hydrogels.

##### 4.2.2.3 X-Ray Scattering

Small- and wide-angle X-ray scattering (SAXS/WAXS) measurements were performed on a SAXSLabs Ganesha pinhole instrument (JJ X-ray System Aps) with an X-ray microsource (Xenocs) and a two-dimensional 300k Pilatus detector (Dectris Ltd., Switzerland). The wavelength, λ, used for all scattering experiments was 1.54 Å, and samples were measured at three sample-to-detector distances. All samples (200 μL) were loaded into a 1.5 mm diameter quartz capillary (Hilgenberg GmbH, Malsfeld, Germany), and the time-averaged scattering intensities were analyzed using a python code. The scattering intensity was corrected to an absolute scale by calibration against water. Supramolecular dimensions were extracted from SAXS data by using the SasView software and fitting the scattering curves to an elliptical-cylinder model (www.sasview.org).

##### 4.2.2.4 Cryo-TEM

Images were obtained at the National Center for High Resolution Electron Microscopy within Lund University on a JEOL JEM-2200 transmission electron microscopy instrument using a TVIPS F416 camera with an accelerator voltage of 200 kV. Samples (4 μL) were vitrified after 3 h of incubation at 21 °C on lacey carbon film-covered copper grids employing a Leica EM GP automatic plunge freezer. Grids were glow-discharged prior to sample preparation. Image processing was performed on the open-source software ImageJ (https://imagej.nih.gov) with the FIJI processing package.

## Supporting Information Available

Experimental details, including materials used, peptide synthesis, and sample preparations; FTIR data of P0–P6 at different pH values; IE FTIR data of ^13^C-labeled sequences (P0–P3); FTIR data of Phe^CN^-labeled P0 peptides; free energy calculations of parallel and antiparallel arrangements, along with AmyloFit modeling of P0–P9; and additional cryo-TEM images, rheology, and SAXS and WAXS data (PDF)

## Standard amino acid one- and three-letter codes are used throughout.

Cryo-TEM: cryogenic-transmission electron microscopy
Fmoc: (*N*-(9-fluorenyl)methoxycarbonyl)
HBTU: *O*-(Benzotriazol-1-yl)-*N,N,N’N’-*tetramethyluronium hexafluorophosphate
HOBt: 1-hydroxybenzotriazole hydrate
HPLC: high-performance liquid chromatography
IE: isotope-edited
MAX: H_2_N–VKVKVKVK–(V^D^PPT)–KVKVKVKV–CONH_2_
MS: mass spectrometry
MDP: multidomain peptide, CH_3_O–XX(SL)_6_XX–CONH_2_, where *X* is K, R, D, or E
NACore: H_2_N–GAVVTGVTAVA–OH
RADA16: CH_3_O–(RADA)_4_–CONH_2_
Phe^CN^: *p-*cyanophenylalanine
PSA: prostate-specific antigen
SAXS and WAXS: small- and wide-angle X-ray scattering
SgI: semenogelin I
TFA^–^: trifluoroacetate
TFA: trifluoroacetic acid
FTIR: Fourier-transform infrared spectroscopy
