# Supplementary material for: Insights into the Hierarchical Assembly of a Chemically Diverse Peptide Hydrogel Derived from Human Semenogelin I
Source: ACS Nano. 2024 Nov 1;18(45):31109–22. doi: 10.1021/acsnano.4c08672 (PMC11562788; doi:10.1021/acsnano.4c08672)
Supplement: Supplementary file 1 — nn4c08672_si_001.pdf [file nn4c08672_si_001.pdf]

Supplementary Information

Insights into the Hierarchical Assembly of a Chemically Diverse Peptide Hydrogel Derived from Human Semenogelin I

Brett H. Pogostin[1,2,*], Kerilyn Godbe[1], Marija Dubackic[2], Isabelle Angstman[1], William Fox[1], Natalie Giovino[1], Matija Lagator[1], Abigail Payson[1], Marisa LaBarca[1], Birgitta Frohm[3], Katja Bernfur[3], Sara Linse[3], Casey H. Londergan[1], Ulf Olsson[2], Luigi Gentile[2,4] and Karin S. Åkerfeldt[1,*]

[1]Department of Chemistry, Haverford College, Haverford, PA 19041, US. [2]Department of Physical Chemistry, Lund University, PO Box 124, SE-221 00 Lund, Sweden. [3]Biochemistry and Structural Biology, Lund University, PO Box 124, SE-221 00 Lund, Sweden. [4]Department of Chemistry, University of Bari Aldo Moro, Via Orabona 4, 70126 Bari, Italy.

FTIR Spectroscopy

Part A.

- IR spectra of TFA and TFA exchanged P3 (to chloride) compared to TFA exchanged (to chloride) P0
- IR spectra of P0-P5, pH 3.0-10.0 (2.0 mM), P6, pH 8 (2.0 mM), and P7-P8, pH 8 (1.0 mM)

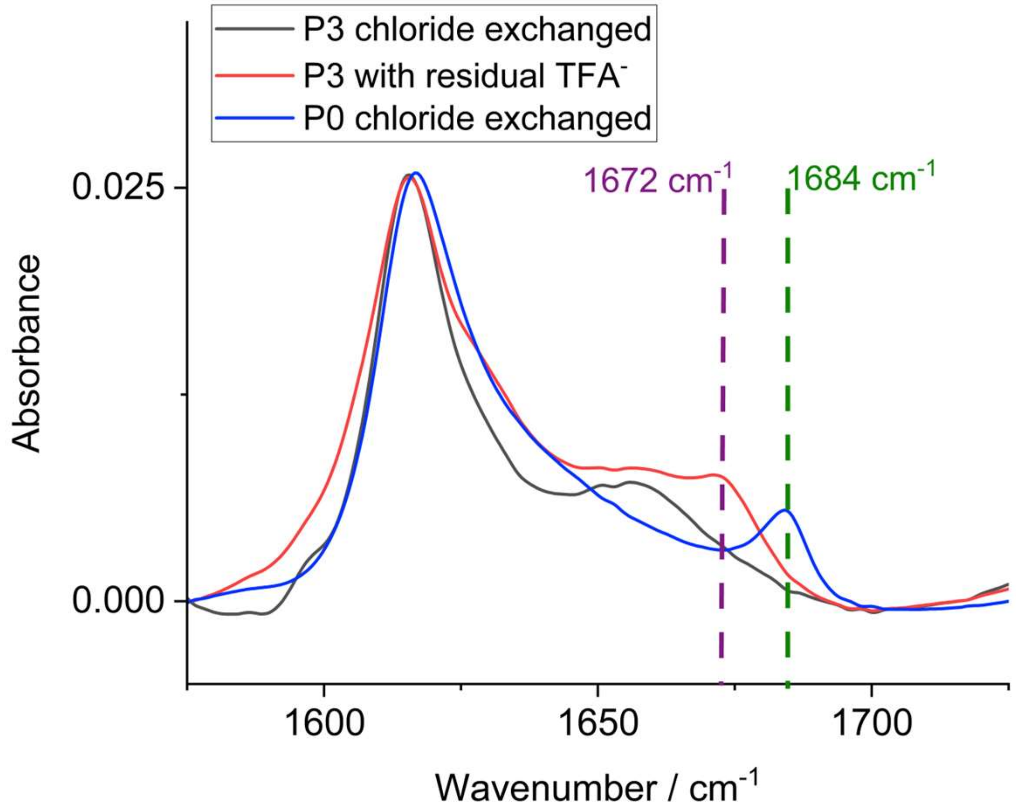

Figure S1. Amide I region IR spectra for chloride-exchanged samples and a sample with HPLC purified residual TFA^- . The weak band at 1672 cm^{-1} in the P3 unexchanged sample (red) exhibits the presence of residual TFA^- as the peptide's negative counterion. P3 after exchange (dark gray) shows no trace of this signal, and P3 has no high-frequency amide I band due to its parallel secondary structure. P0 after ion exchange (blue) shows an amide I signal at 1684 cm^{-1} consistent with its antiparallel structure.

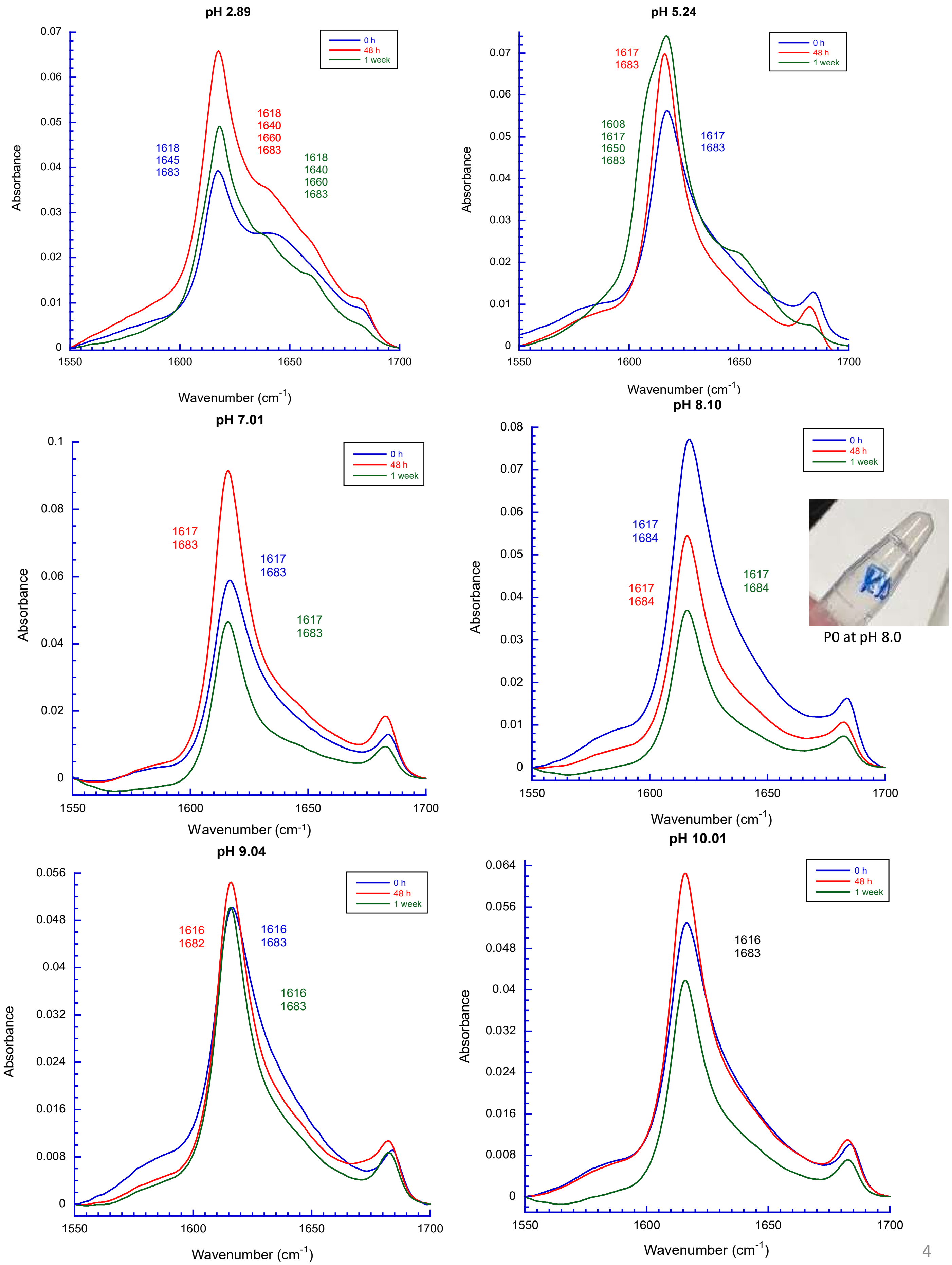

Figure S2. P0 (2.0 mM in D_2O) at pH 2.89, 5.24, 7.01, 8.10, 9.04 , 10.01 at 0 h (blue), 48 h (red) and 1 week (green).

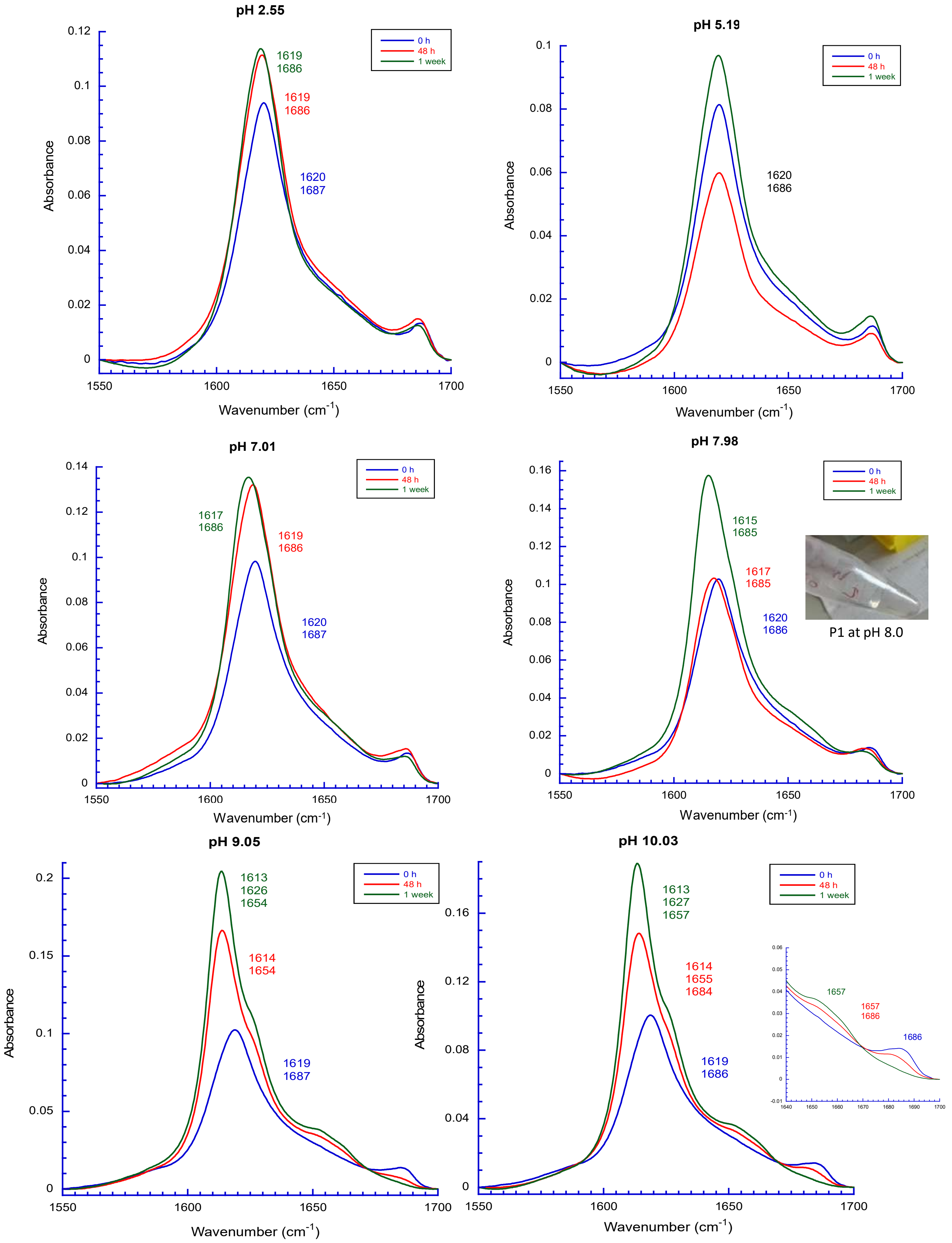

Figure S3. P1 (2.0 mM in D_2O) at pH 2.55, 5.19, 7.01, 7.98, 9.05 , 10.03 at 0 h (blue), 48 h (red) and 1 week (green). Insert shows disappearance of the high-frequency band over time at pD 10.03.

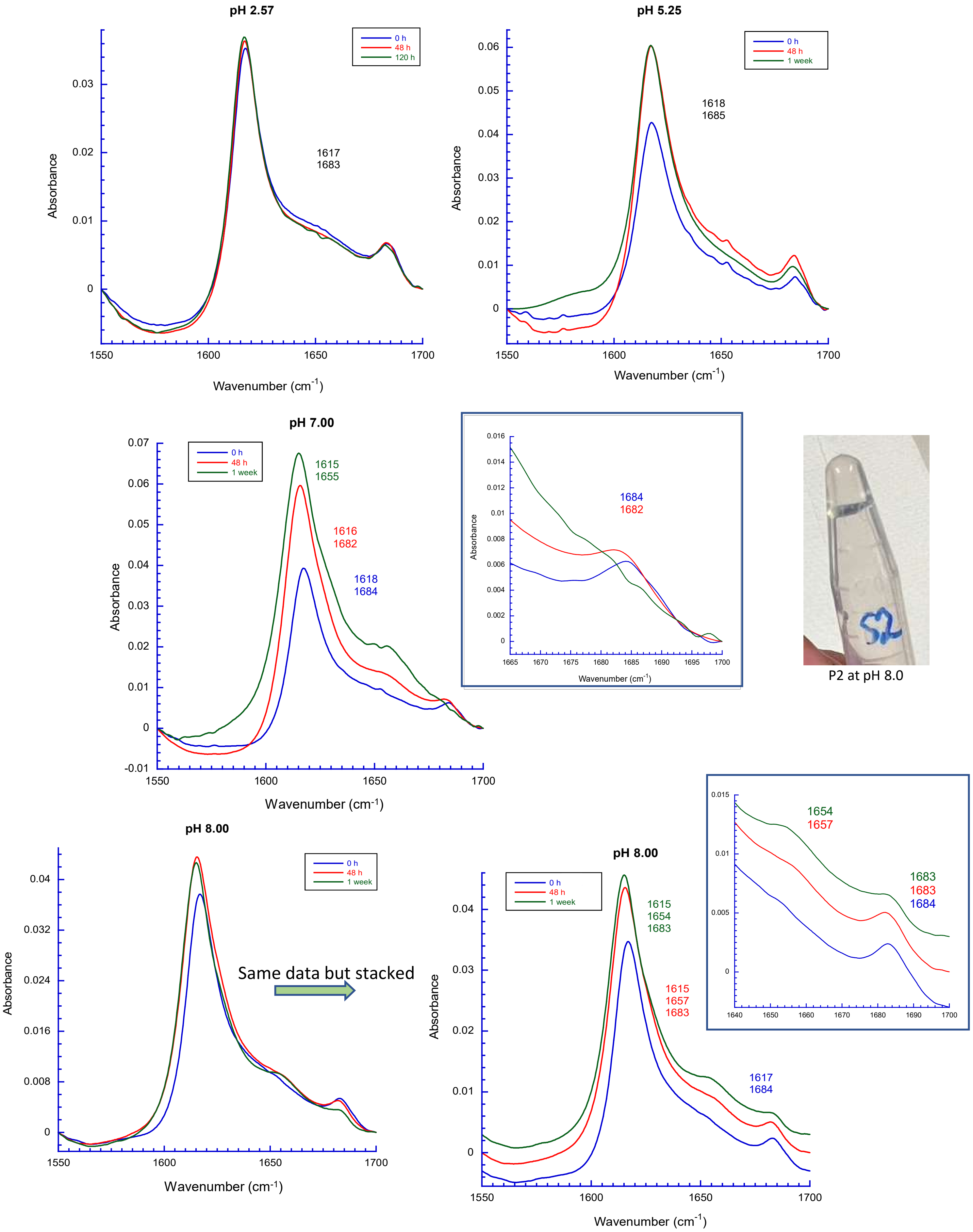

Figure S4. P2 (2.0 mM in D_2O) at pH 2.57, 5.25, 7.00, 8.00 at 0 h (blue), 48 h (red) and 1 week (green). Inserts show disappearance of the high-frequency band over time at pD 7.00 and pD 8.00.

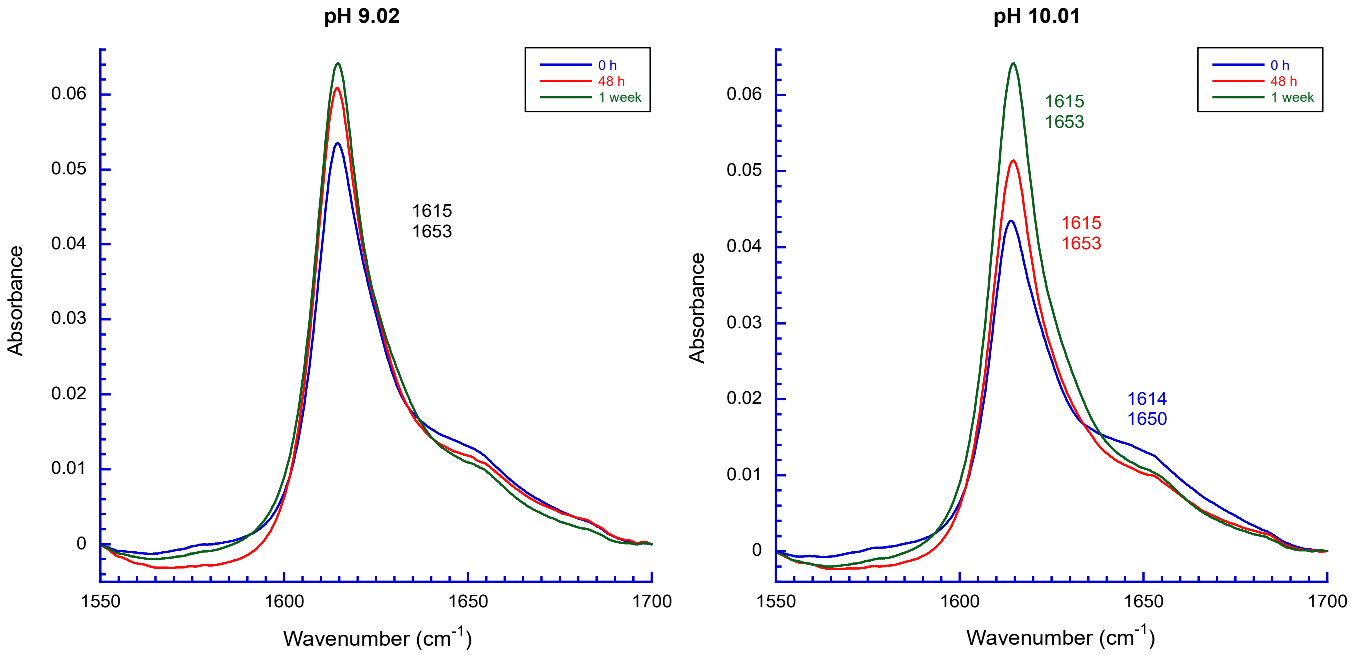

Figure S4 (continued). P2 (2.0 mM in D_2O) at pH 9.02 , 10.01 at 0 h (blue), 48 h (red) and 1 week (green).

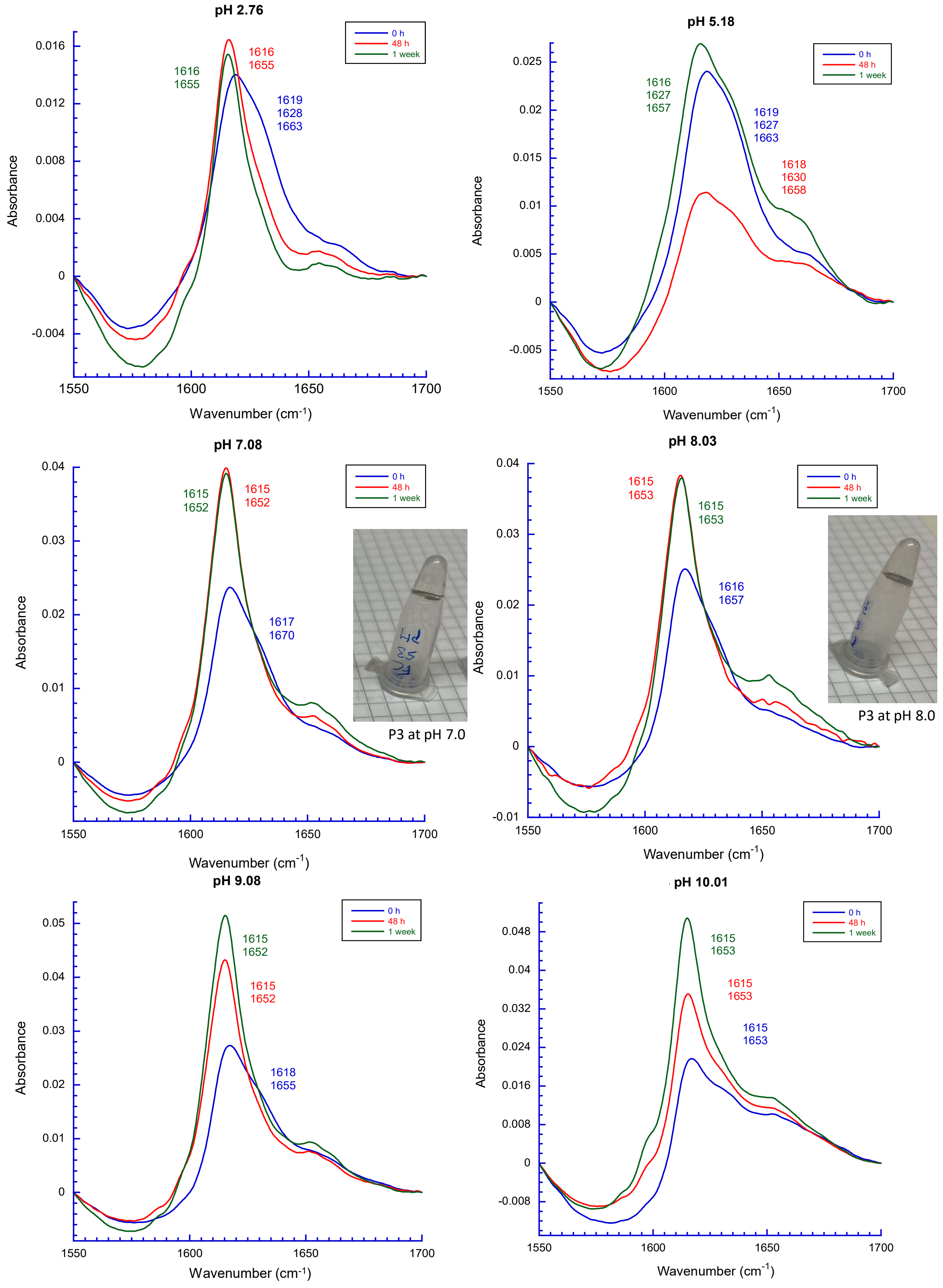

Figure S5. P3 (2.0 mM in D_2O) at pH 2.76, 5.18, 7.08, 8.03, 9.09 , 10.15 at 0 h (blue), 48 h (red) and 1 week (green).

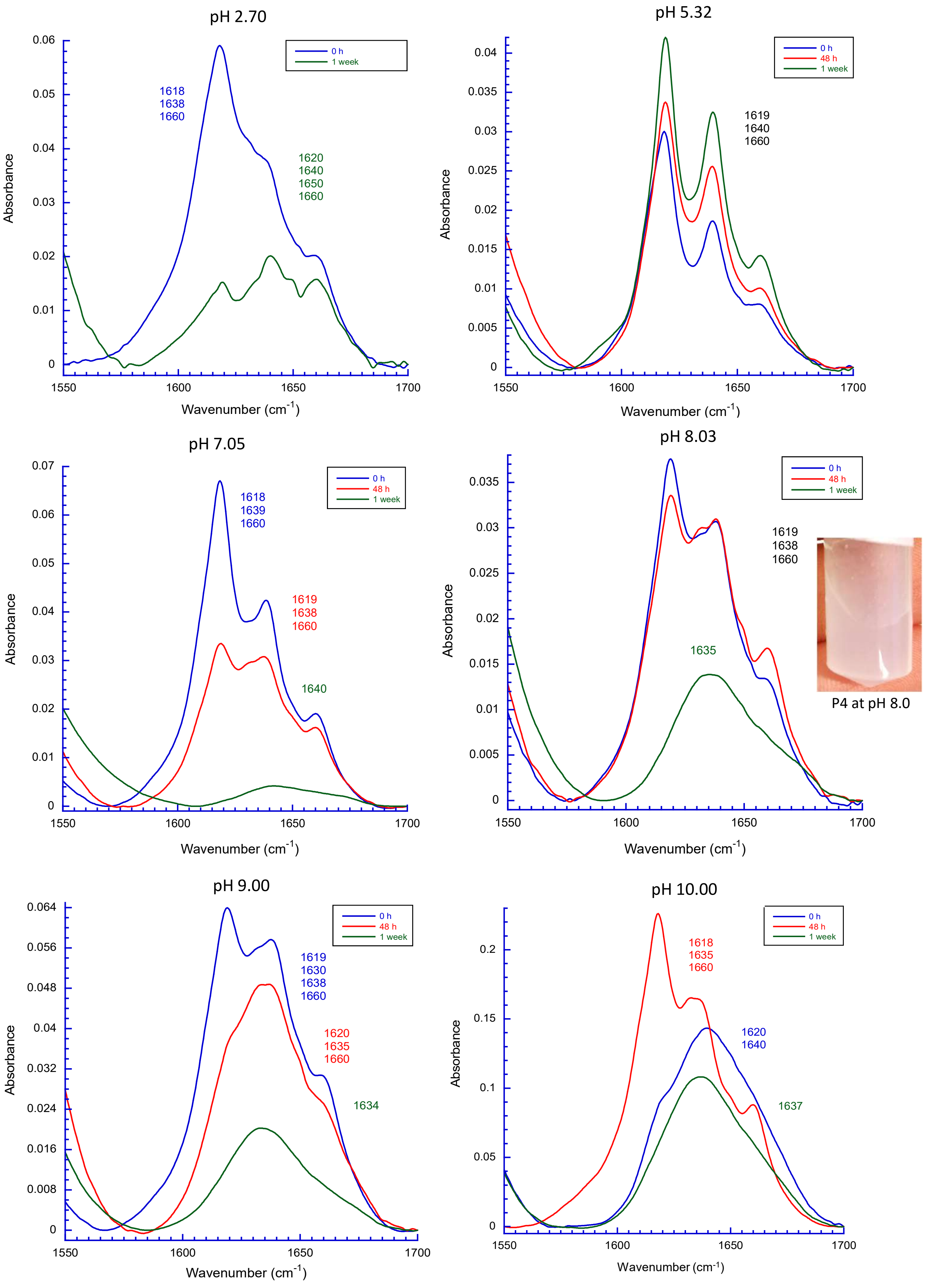

Figure S6. P4 (2.0 mM in D_2O) at pH 2.70, 5.32, 7.05, 8.03, 9.00, 10.00 at 0 h (blue), 48 h (red) and 1 week (green). The initially formed hydrogel at pH 8 is colloidally unstable and undergoes phase separation (see image).

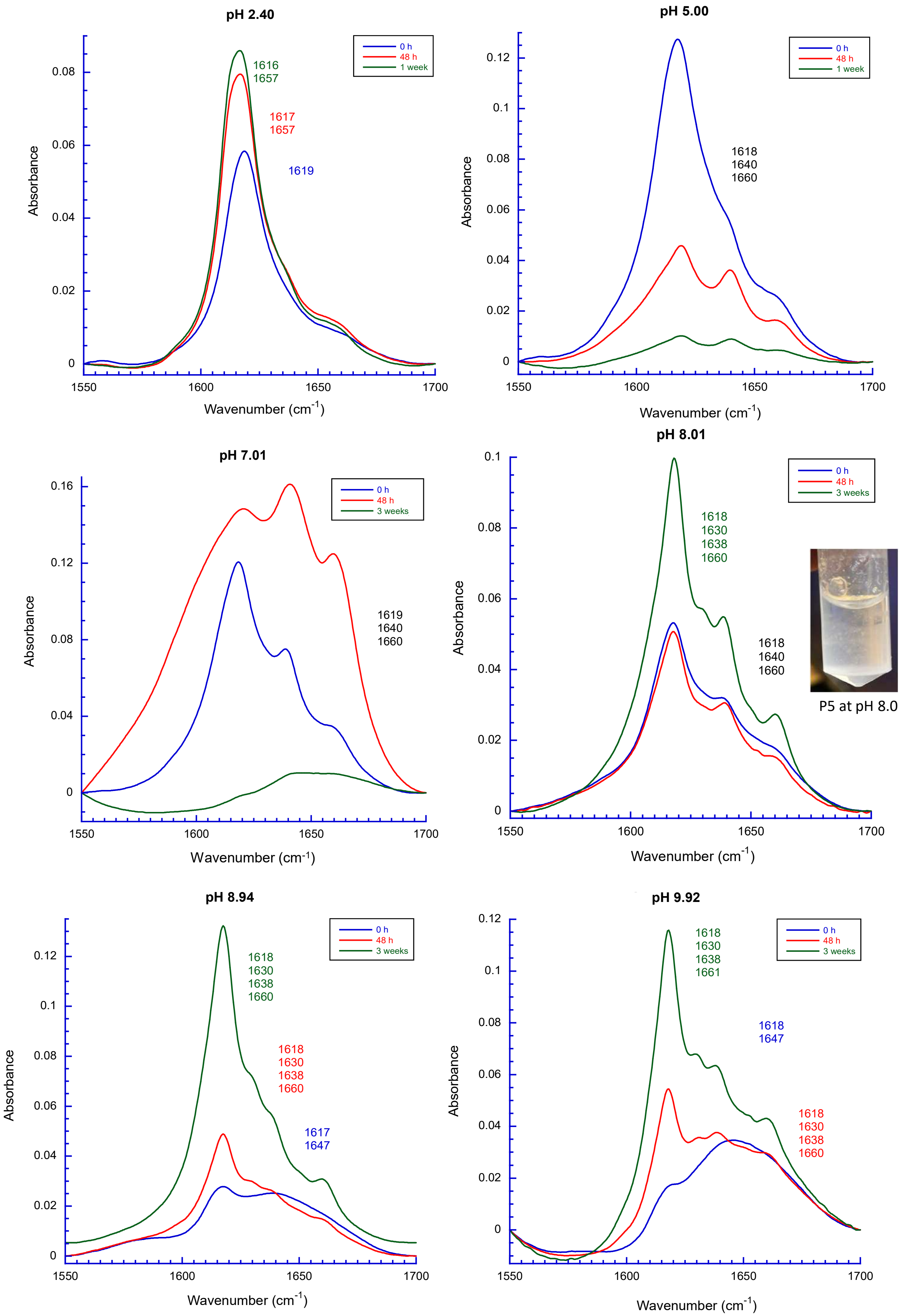

Figure S7. P5 (2.0 mM in D_2O) at pH 2.40, 5.00, 7.01, 8.01, 8.94 , 9.92 at 0 h (blue), 48 h (red) and 1 week (green). The initially formed hydrogel at pH 8 is colloidally unstable and undergoes phase separation (see image).

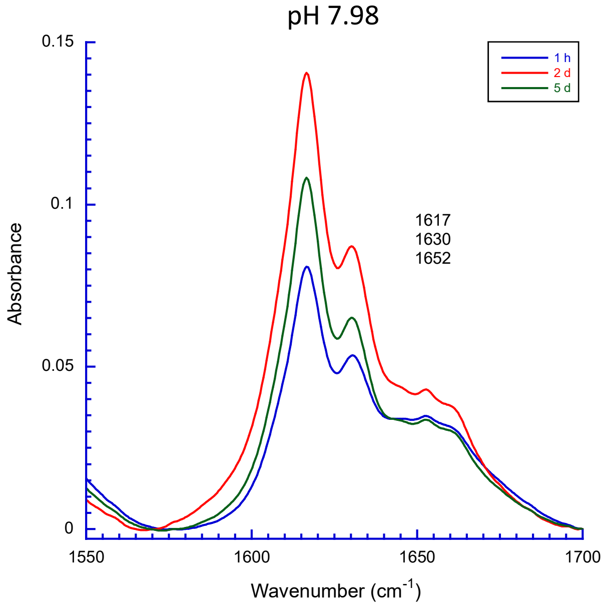

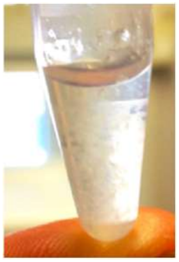

P6 at pH 8.0

Figure S8. P6 (2.0 mM in D_2O) at pH 7.98 at 0 h (blue), 48 h (red) and 1 week (green). The initially formed hydrogel at pH 8 is colloidally unstable and undergoes phase separation (see image).

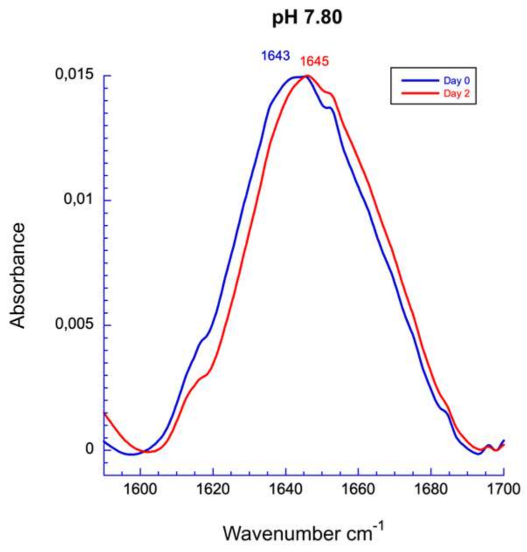

Figure S9. P7 (1.0 mM in D_2O) at pH 7.80 at day 0 (blue) and day 2 (red).

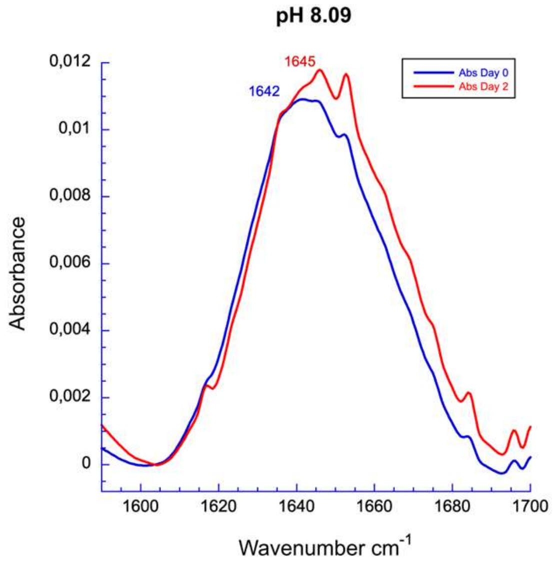

Figure S10. P8 (1.0 mM in D_2O) at pH 8.09 at day 0 (blue) and day 2 (red).

P0-P8: Summary of FTIR Studies

From the ^{13}C-labeling experiments on P0-P3 (SI Part B, below), we have verified that the presence of the IR signal at 1685 cm^{-1} indicates an antiparallel β–strand arrangement.

Summary (P0)

- At ca. pH 5 and lower, the signal at 1685 cm^{-1} decreases in intensity over time, indicating a reorganization from an antiparallel to a parallel strand arrangement within the fibril sheet.
- Interestingly, at pH 2.9 two signals develop at 1640 and 1660 cm^{-1}, resembling those observed in the spectra obtained for P4, P5, and P6.

Summary (P1)

- At pH 2.5-8.0, the data is consistent with antiparallel β–sheet. At pH 8.0, you start to see a possible decrease at 1685 cm^{-1} over time. This becomes more noticeable at higher pH, where this signal disappears over 1 week, indicating a strand reorientation to parallel.
- Concurrently with the disappearance of 1685 cm^{-1}, a signal at 1660 cm^{-1} is increasing, similar what is observed for P0 at low pH, and also seen in spectra of P4, P5, and P6.

Summary (P2)

- At pH 5 and below, P2 forms antiparallel sheets.
- At pH 7-8 a transition is observed from antiparallel to parallel.
- Only parallel sheets are seen at above pH 8.

Summary (P3)

- Only parallel strand orientation is observed at pH 3-10, as the 1685 cm^{-1} signal is absent.
- The spectral data provide the sharpest signals at pH 7-9.

Summary (P4)

- A parallel strand orientation is observed at pH 3-10 as the 1685 cm^{-1} signal is absent.
- The spectral features at 1640 and 1660 cm^{-1} are very interesting and due to their sharpness, are likely indicative of structurally well-defined interactions.
- The peptide undergoes phase separation.
- The β–sheet structure is lost within 1 week as shown by the development of a broad signal at around 1650 cm^{-1}, consistent with random coil.

Summary (P5)

- A parallel strand orientation is observed at pH 3-10.
- The spectral features at 1640 and 1660 cm^{-1}, due to their sharpness, are likely a result of a well-defined structure.
- At pH 2.4, a clean β-sheet structure is observed that remains. At other pH values, the β–sheet is lost to generate a random coil, as evidenced by a broad signal at 1650 cm^{-1}.
- P5 undergoes phase separation.

Summary (P6)

- P6 forms a hydrogel at at 2.0 mM, pH 8.0.
- P6 is colloidally unstable and undergoes phase separation.

Summary (P7)

- P7 is unstructured at 1.0 mM, pH 8. Due to low solubility, it was not tested at higher concentrations.

Summary (P8)

- P8 is unstructured at 1.0 mM at pH 8. Due to low solubility, it was not tested at higher concentrations.

pH 7.0

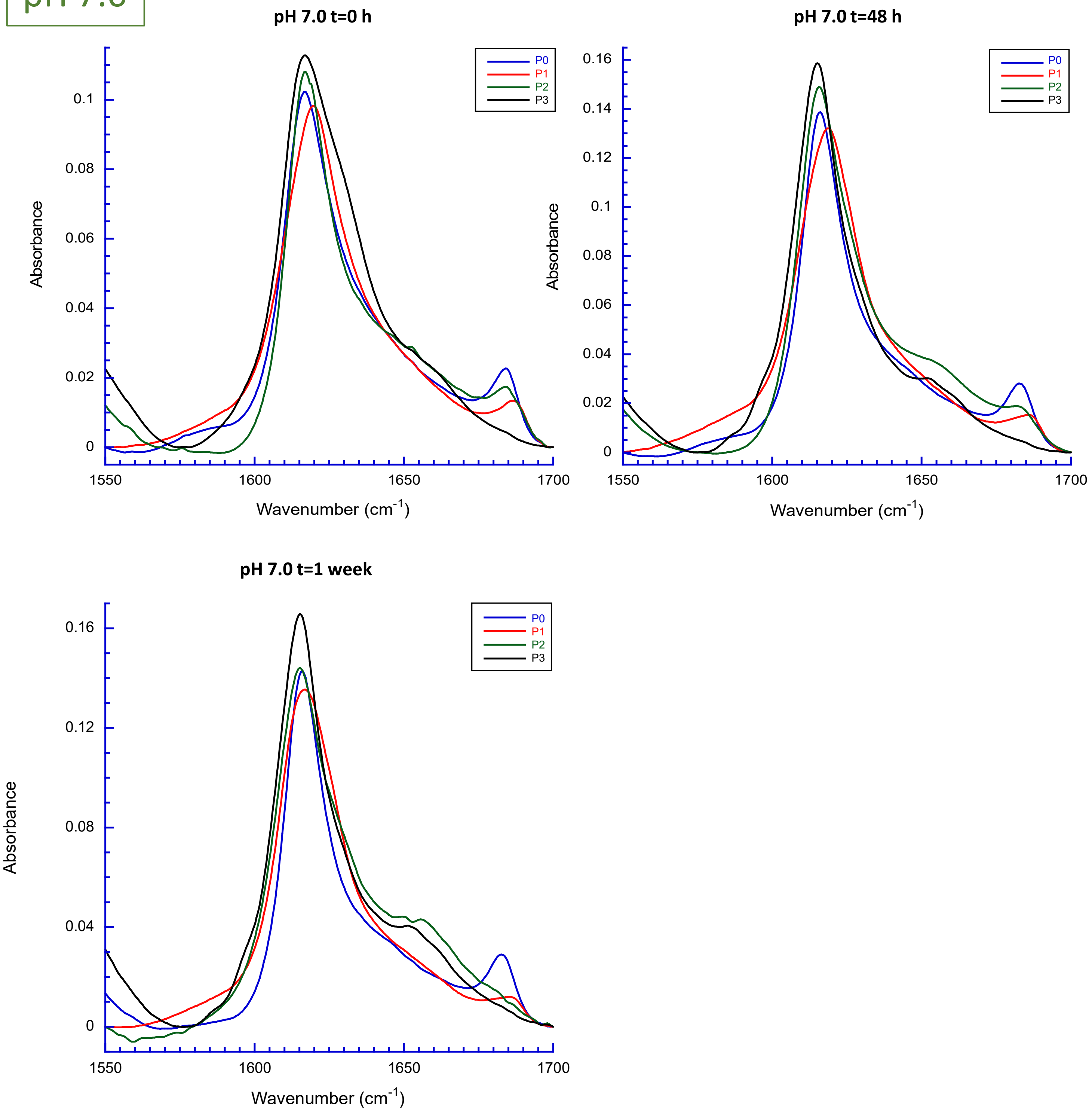

Figure S11. Overlayed IR spectra at pH 7.0 of P0-P3, as indicated, at 2.0 mM in D_2O and at 0 h, 48 h, and 1 week of incubation time at ambient temperature. The signal at 1685 cm^{-1} remains for P0, is absent for P3, and decreases over time for P1 and P2, more so for P2 than P1. The signal decrease is also accompanied by loss of β–sheet structure, as indicated by an increase in 1650 cm^{-1} (random coil).

pH 8.0

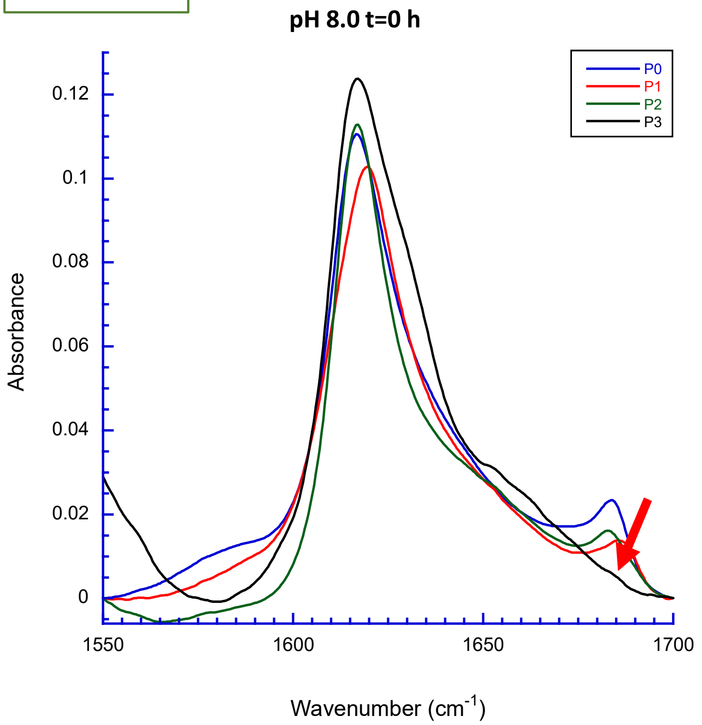

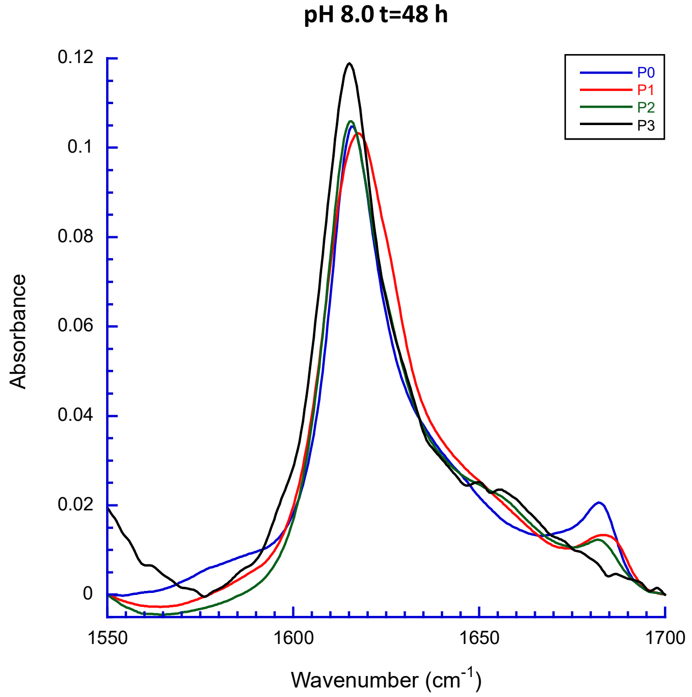

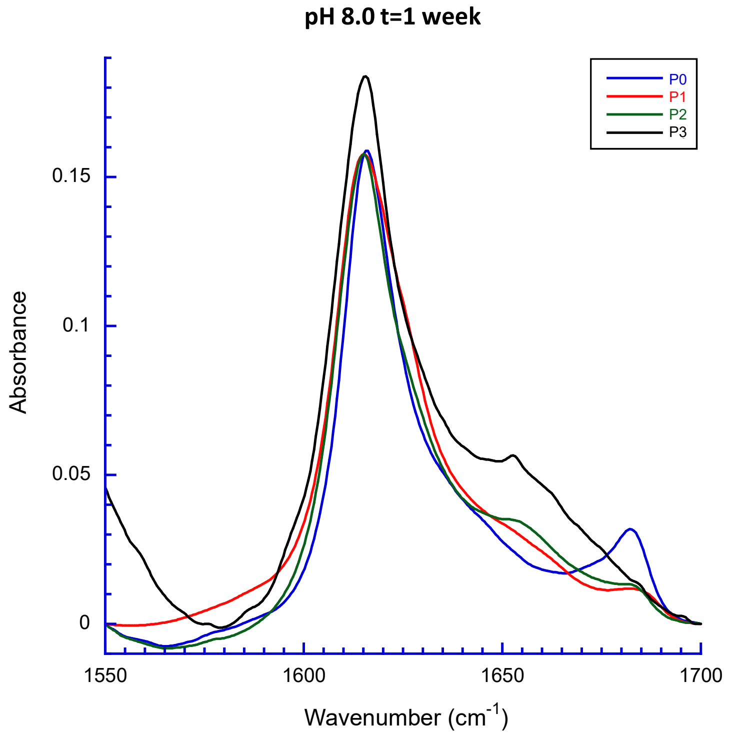

Figure S12. Overlayed IR spectra at pH 8.0 of P0-P3, as indicated, at 2.0 mM in D_2O at 0 h, 48 h, and 1 week of incubation time at ambient temperature. The signal at 1685 cm^{-1} remains for P0, is absent for P3, and decreases over time for P1 and P2.

FTIR Spectroscopy

Part B. IE FTIR spectra of P0-P3

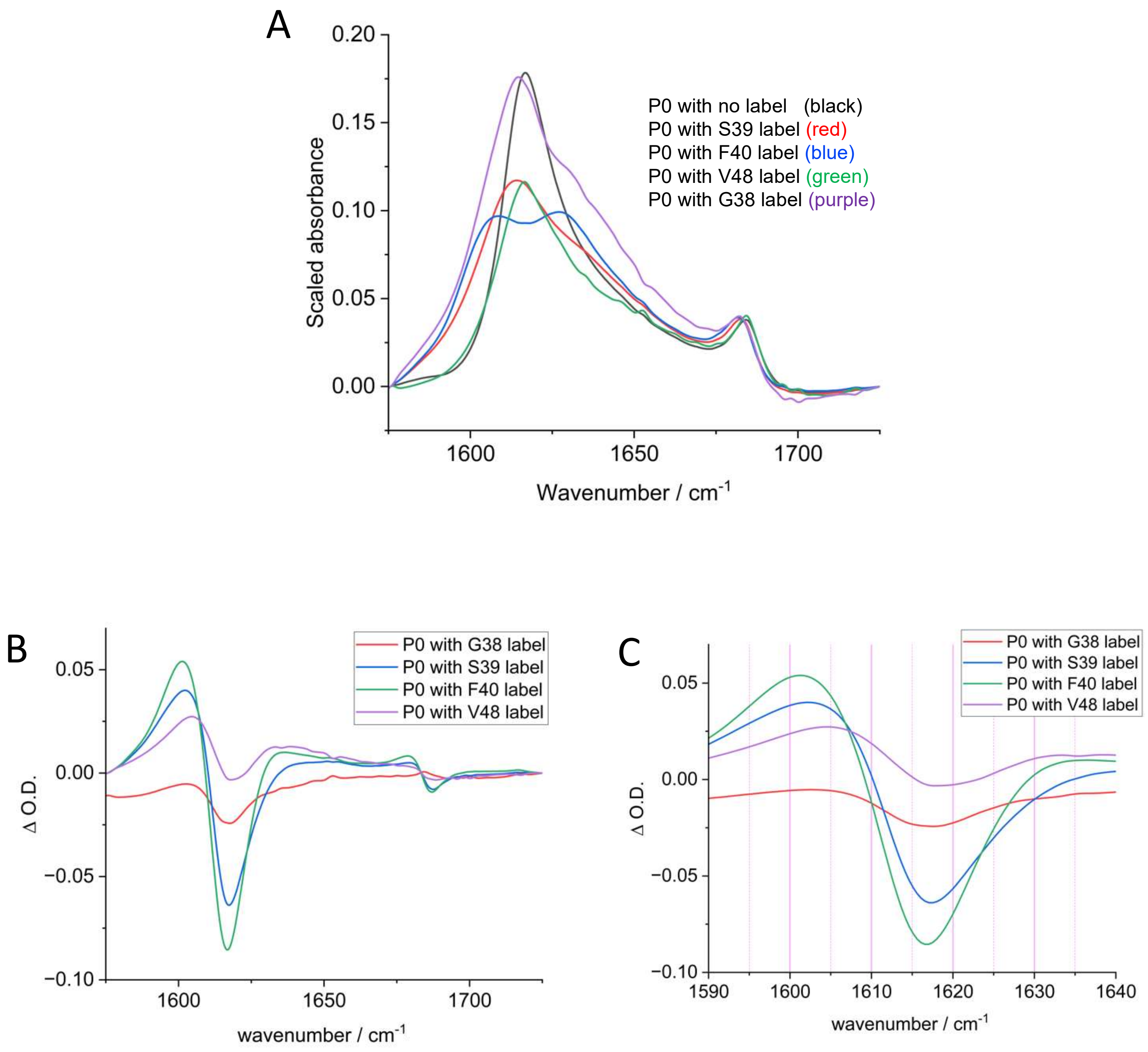

Figure S13. (A) Amide I IR absorption spectra in D_2O for P0 peptides, normalized to the intensity of the high-frequency band. **(B-C)** Difference spectra for ^{13}C-labeled P0 peptide samples *vs* the unlabeled P0 spectrum in **A**. Each difference spectrum shows the quantitative changes to the overall spectrum due to the single ^{13}C isotopic substitution with **(B)** full amide I region and **(C)** zoomed in to the low frequency region to more clearly slow varying quantitative shifts of the low frequency absorptions. Negative features indicate that spectral intensity was lost due to the ^{13}C isotopic substitution: positive features indicate new spectra intensity introduced by the substitution. For a discussion, see next page.

Figure S13: Discussion

The isotopic shift observed in the low-frequency difference spectra (focused on the band that in unlabeled P0 is centered at about 1617 cm^{-1}) is *much smaller* than the ~35 cm^{-1} expected for a single isotopic substitution. This speaks to the delocalized nature of the normal modes that contribute to that spectral region and means that *all* of these residues are strongly coupled to ^{12}C amide I groups to different extents depending on their position in the sequence.

The extent of intensity borrowing by the new, lower-frequency exciton normal mode that is introduced by each ^{13}C isotopic substitution varies across the four sites. This leads to the conclusion that all four of these label sites participate in the β–sheet (at least part of the time). The S39 and F40 labels are the most strongly coupled to ^{12}C amide I's (thus they are fully enclosed "in the sheet"). In contrast, the G38 and V48 labels are much less strongly coupled, suggesting that they are either in the sheet and at the edges (and thus coupled to fewer ^{12}C neighbors by virtue of the topology) or spending part of their time outside the sheet, in which case their signals would be much weaker and broader and not as clearly evident in the difference spectra.

In all amide I spectra, especially for the strongly coupled S39 and F40 label sites, the high frequency mode at ~1684 cm^{-1} also shifts to slightly lower frequency due to the isotopic substitution. This provides evidence that the isotopic substitutions change the frequencies of all the observed normal modes (because they are all delocalized), but the frequency shift is *very small* (on the order or -1 cm^{-1}) due to the minor participation of the ^{13}C labels in that specific normal mode (as opposed to their over-representation in the low-frequency exciton modes). This small shift of the high frequency bands provides further evidence that they are delocalized amide I vibrations and are not coming from some other molecular feature (i.e. TFA anion impurity, see Figure S1, or some sort of salt bridge to lysine, among other possibilities).

The main outcomes of the analysis (a) all of the label sites participate in coupling to ^{12}C amides, consistent with an antiparallel structure for P0 and where the low-frequency (1618 cm^{-1} and below) shifts and different intensities point to their location in the β–sheets, and (b) the high-frequency (1684 cm^{-1}) bands observed here are from amide I modes of an antiparallel structure.

K	G	S	F	S	I	Q	Y	T	Y	H	V	D
37	38	39	40	41	42	43	44	45	46	47	48	49

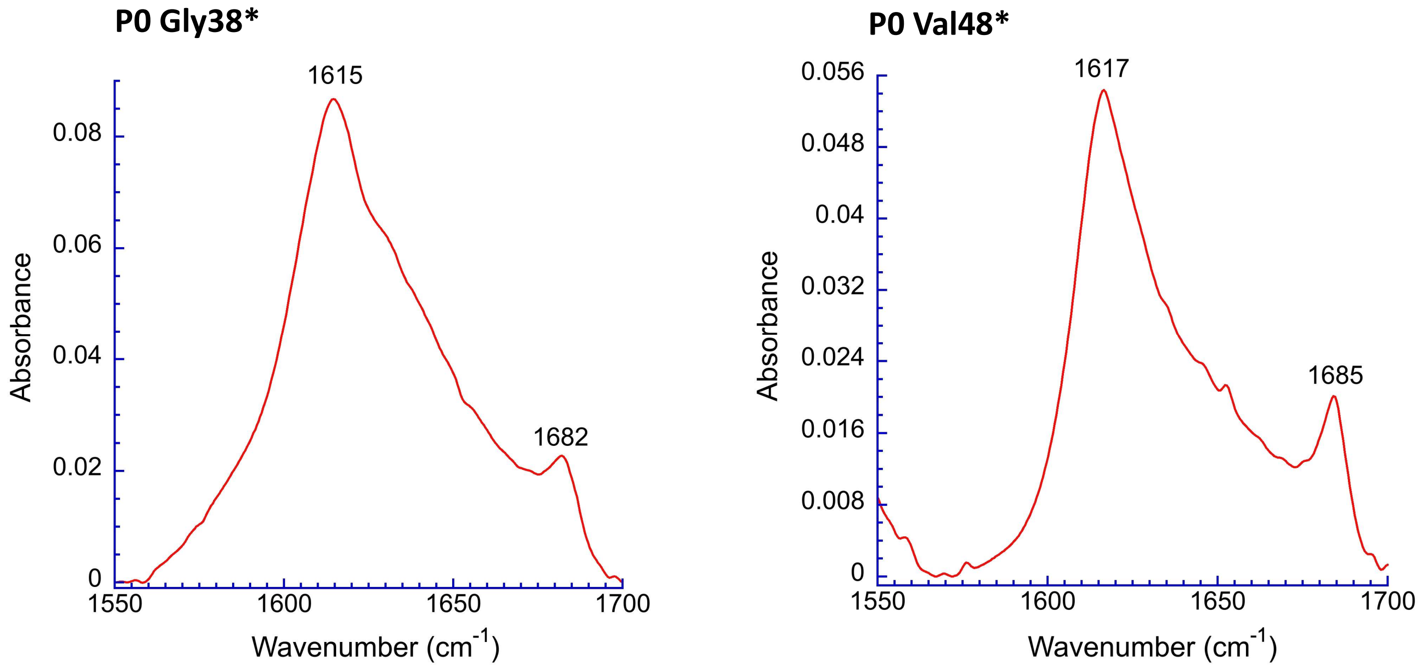

Figure S14. IE FTIR spectra of P0 (2.0 mM in D_2O, pD 8.0) after 24 h (P0 Gly38*) and 5 days (P0 Val48*) of incubation at ambient temperature with an intrinsic vibrational probe, $^{13}C=O$, placed in the backbone amide, at the residue indicated with an asterisk (Figure 1C). These spectra look very similar to that of P0 Ser39* (Figure 3).

K	G	S	F	S	I	Q	Y	T	Y	H	V	D
37	38	39	40	41	42	43	44	45	46	47	48	49

24 h, Day 6

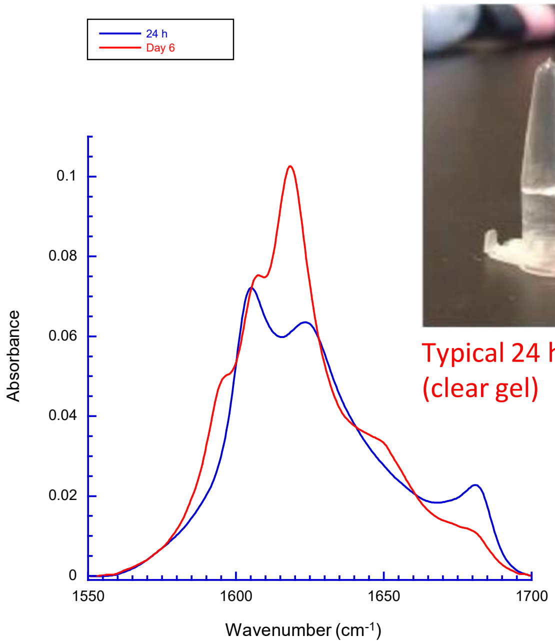

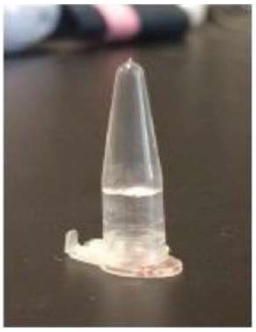

Typical 24 h sample (clear gel)

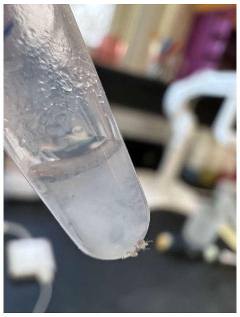

Day 6 sample (with precipitate)

Figure S15. IE FTIR spectra of P0 Ser41* (2.0 mM in D_2O, pD 8.0) incubated at ambient temperature, with sample removed at the times indicated. Images of 24 h (clear gel) and Day 6 (mix of clear gel and precipitate) samples shown.

Summary P0 Ser41* strand reorientation:

- At 24 h: antiparallel.
- At Day 6: mix of antiparallel and parallel with parallel dominating

A.

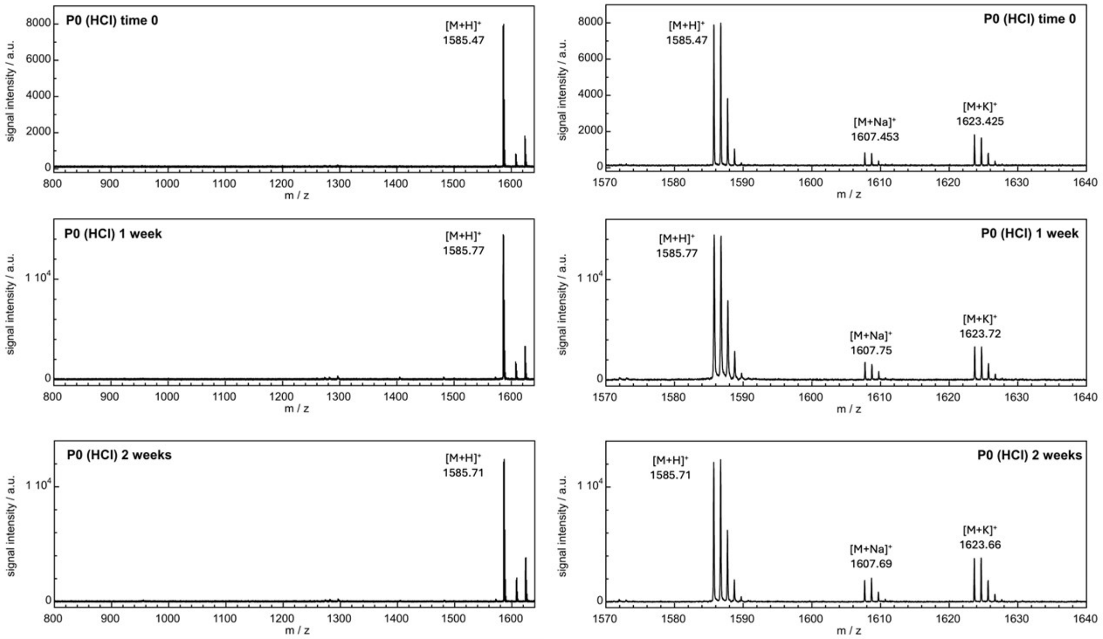

B.

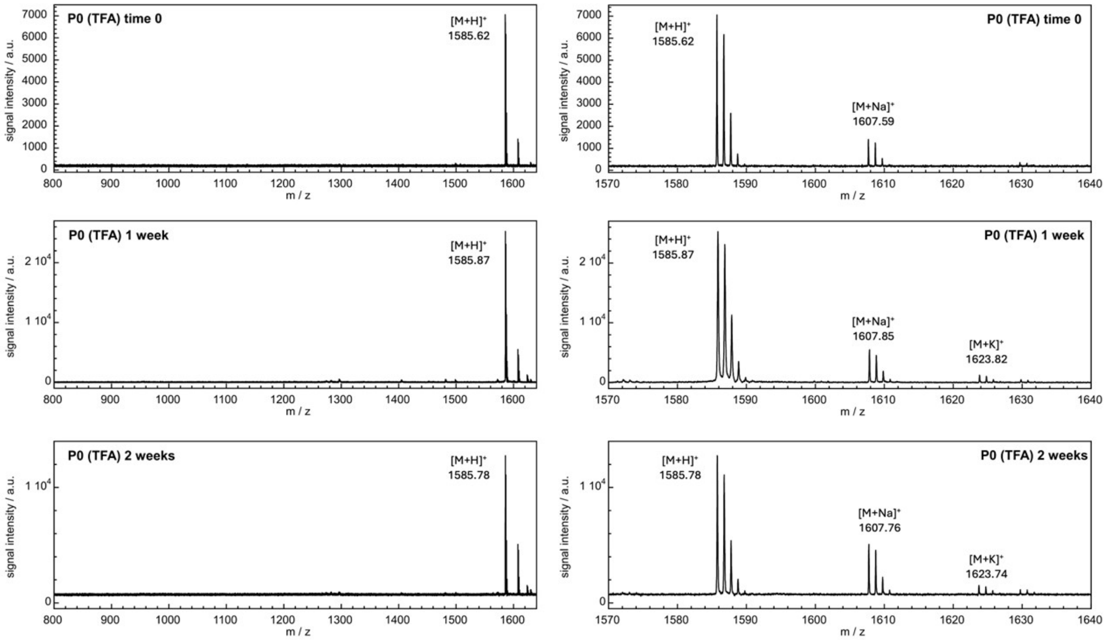

Figure S16. P0 gel stability was evaluated by MALDI-TOF analysis. Two samples were prepared with different counter anions, the P0 chloride salt, which was used in the IR experiments, and the trifluoroacetate salt, which was used in all other studies, unless otherwise indicated. **(A)** A sample of P0 in H_2O at pH 8.0, chloride salt (1.0 mM) and **(B)** trifluoroacetate salt (0.5 mM) were prepared for stability and analyzed by MALDI-TOF mass spectrometry. A soft gel immediately formed upon pH adjustment, after which the samples were incubated at ambient temperature. No appreciable, if any, degradation was observed over 2 weeks. Left panels show the *m/z* range 800-1640 and the right panels a zoomed in view between *m/z* 1570-1640. The calculated mass for P0 $[M+H]^+$ *m/z* = 1585.72.

K	G	S	F	S	I	Q	Y	T	Y	H	V	D
37	38	39	40	41	42	43	44	45	46	47	48	49

P0 Ser41*

S	I	Q	Y	T	Y	H	V	D
41	42	43	44	45	46	47	48	49

P3 Ser41*

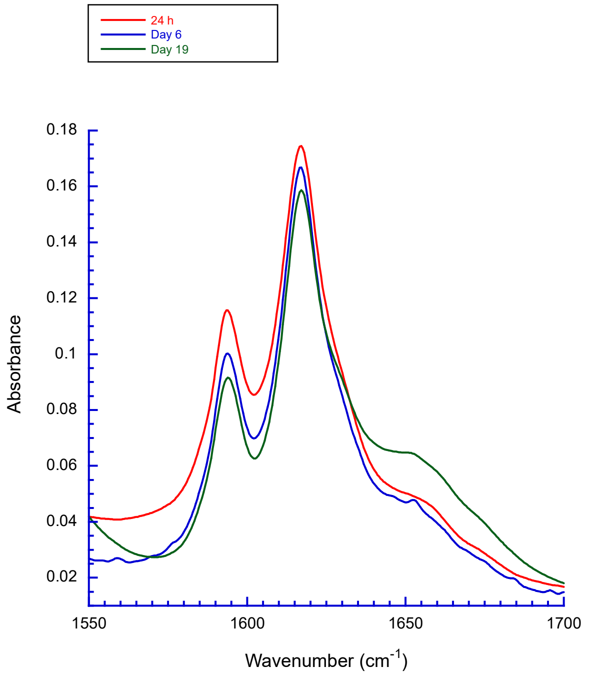

All time points: 1595 + 1617 + shoulder at 1625 + bump at 1655 cm^{-1} parallel

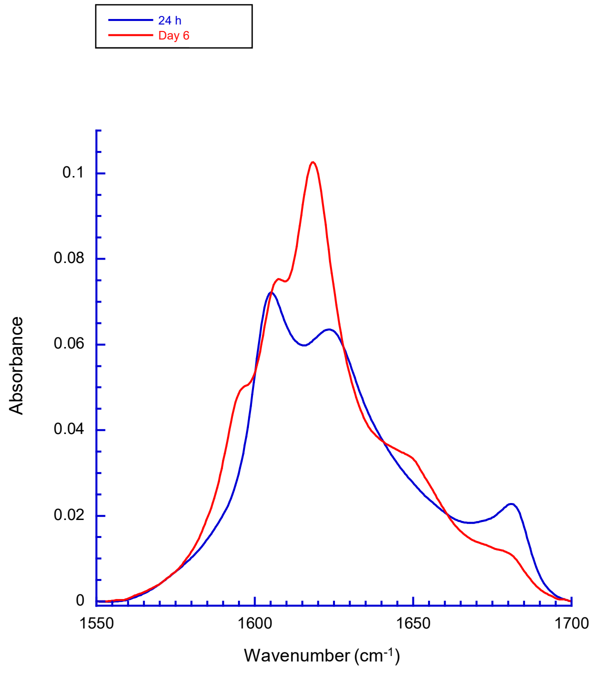

24 h (BLUE): 1605 + 1618 + 1682 cm^{-1}: antiparallel (+ hump around 1655 cm^{-1})
Day 6 (RED): see an additional signal at 1595 cm^{-1} parallel with 1682 lower in intensity

Figure S17. A side by side comparison of IE FTIR spectra of P3 Ser41* (left) and P0 Ser41* (right), both at 2.0 mM in D_2O, pD 8.0, incubated at ambient temperature, with sample removed at the times indicated.

P2 Ser41*

S	F	S	I	Q	Y	T	Y	H	V	D
39	40	41	42	43	44	45	46	47	48	49

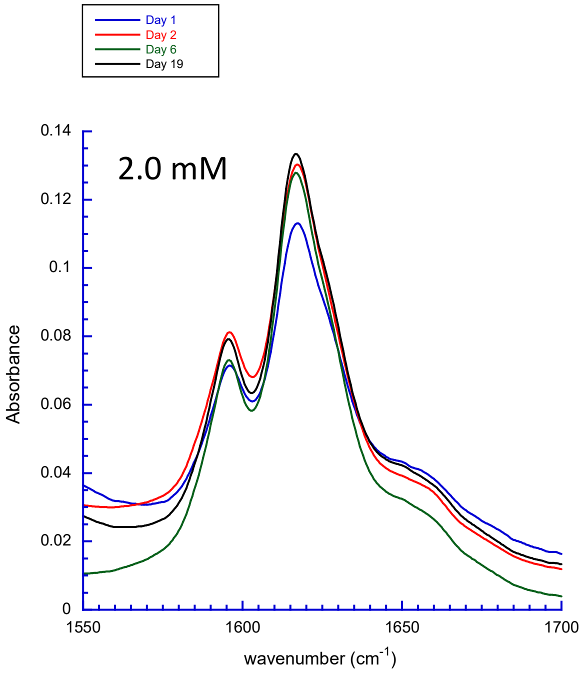

1595 + 1617 + shoulder at 1625 + bump at 1658 cm^{-1} parallel

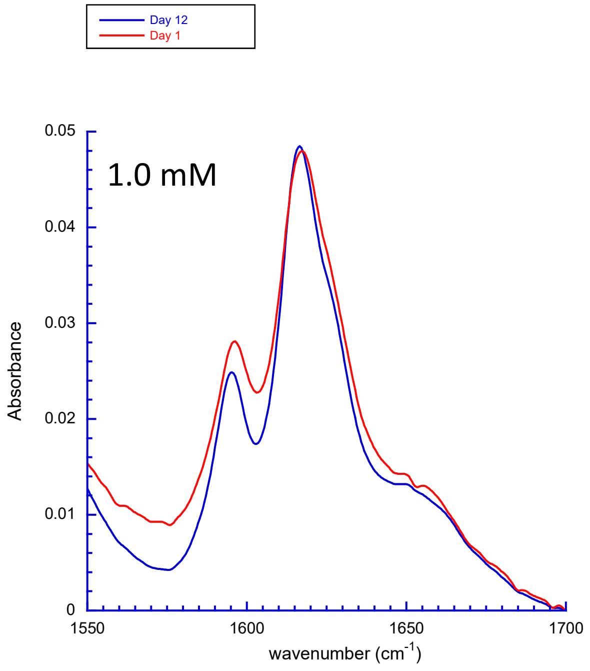

1595 + 1617 + shoulder at 1625 + bump at 1655 cm^{-1} parallel

Figure S18. A side by side comparison of IE FTIR spectra of P2 Ser41* at 2.0 mM (left) and 1.0 mM in D_2O (right) pD 8.0 incubated at ambient temperature, with sample removed at the times indicated.

G	S	F	S	I	Q	Y	T	Y	H	V	D
38	39	40	41	42	43	44	45	46	47	48	49

P1 Ser41*

1.0 mM

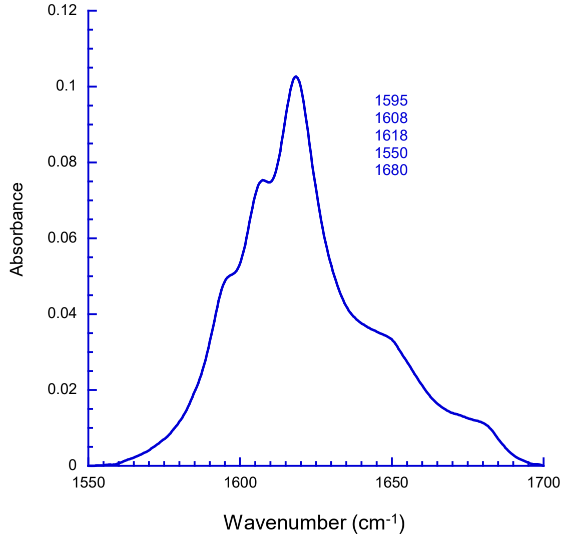

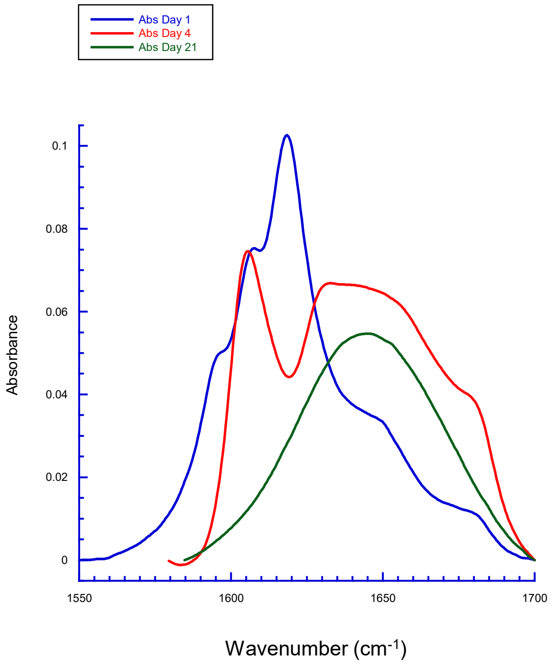

2.0 mM

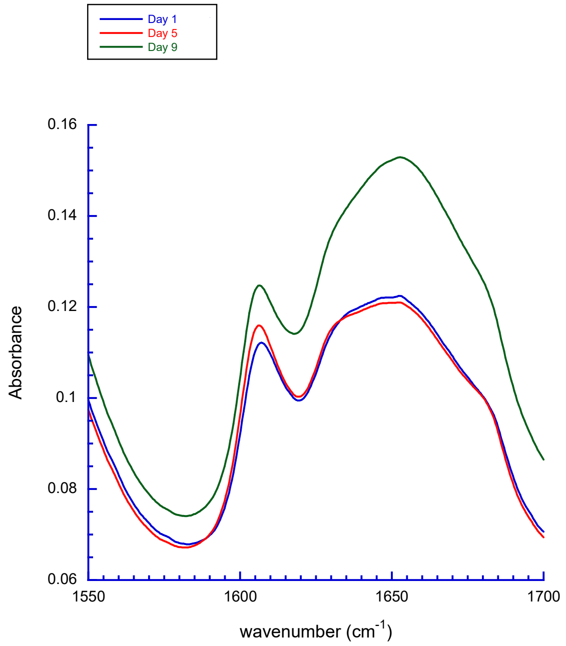

Figure S19. A comparison of IE FTIR spectra of P1 Ser41* at 1.0 mM (top) and at 2.0 mM (bottom), both in D_2O pD 8.0, incubated at ambient temperature, with sample removed at the times indicated.

Summary

- Labeled P1 (P1 Ser41*) shows a mixture of parallel and antiparallel sheet orientations at 1.0 mM. At 2.0 mM, P1 Ser41* only shows the presence of antiparallel strands in combination with unstructured material.
- For unlabeled P1 (Figure S3), as is the case for P0, P2, and P3, the parallel arrangement appears to be thermodynamically more stable than the antiparallel orientation. The IE IR results (Figure S19), however, are less clearcut as they first show a mixture of parallel and anti-parallel sheets (1.0 mM) where the parallel appears to unravel before the antiparallel structure. The results for unlabeled P1 are consistent with a kinetically formed antiparallel structure that over time, particularly at high pH (9 and 10, but also at 7 and 8 more slowly), form a more stable parallel β–sheet. Taken together, we interpret these results to mean that the antiparallel and parallel arrangements are more similar in thermodynamic stability than for P0, P2 and P3.
- P1 produces a less stable gel than P0, P2 and P3, as noticed in handling. While it initially forms a nice robust clear gel, it breaks up more easily than the gels generated by P0, P2 and P3. The IE IR data confirms that the structure at pD 8.0 is lost over time.

FTIR Spectroscopy

Part C. Free energy calculations and Amylofit modeling

Calculation using tabulated pair-potentials.

The inter-residue potentials at interfaces were taken from Table 1 of Keskin et al. 2009.[55] First, we calculated the residue-mediated sidechain contributions to the free energy of dimers of P0-P10 for the two cases of in-register parallel and in-register antiparallel arrangement. For most peptides, the difference between those two arrangements is less than 1 kT (2.5 kJ/mol), in other words negligible (Figure S20A and B). However, P1, and maybe also P3 and P5, display slightly larger differences in favor of parallel arrangement. Second, we used the solvent-mediated potentials,[55] which take into account the burial of the side-chains, for the two cases of in-register parallel and in-register antiparallel arrangement. For most peptides, the difference between those two arrangements is still less than 1 kT (Figure S20C and D). Again, we find that P1, and maybe also P0, P3 and P5, display slightly larger differences in favor of a parallel arrangement.

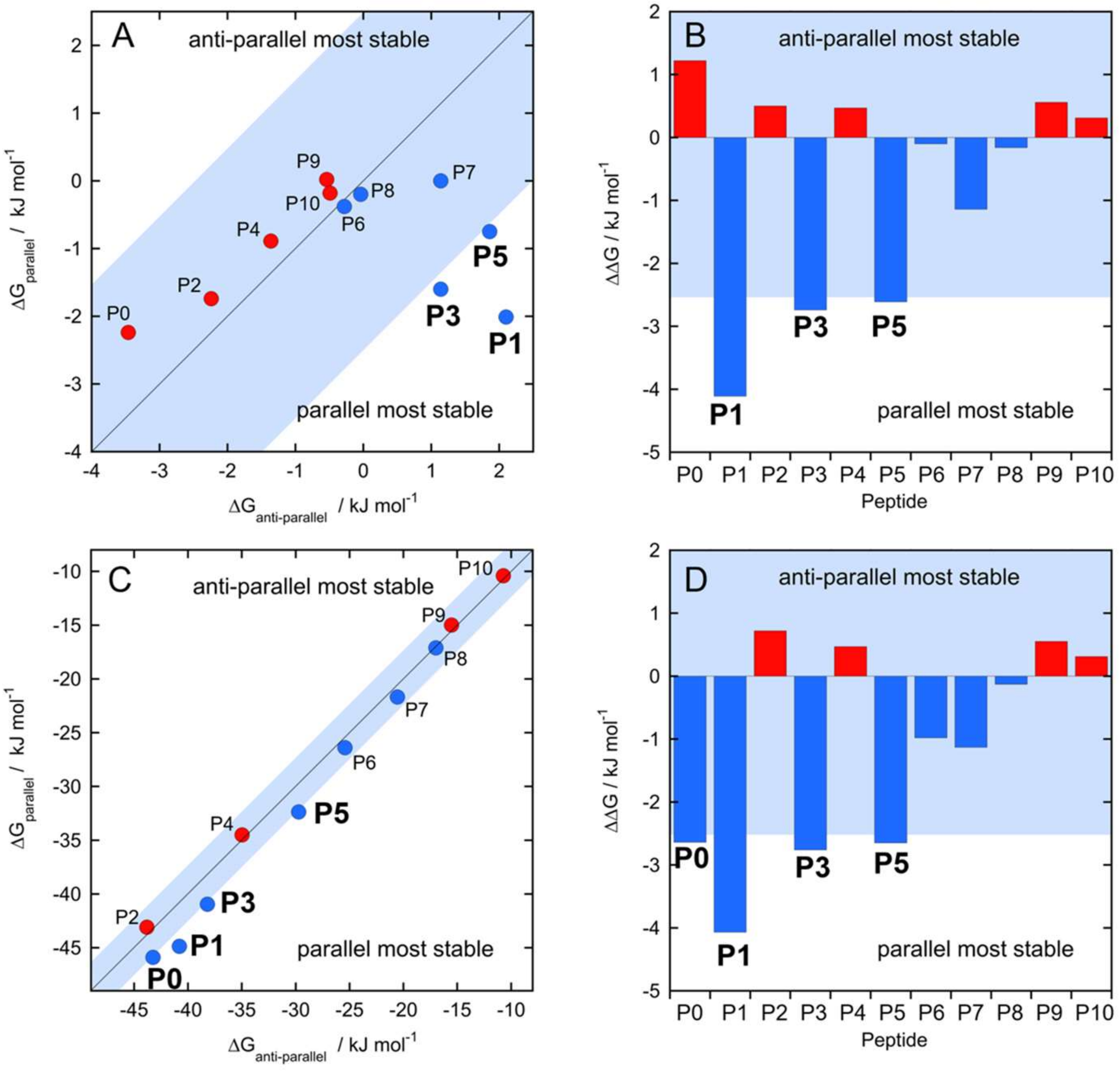

Figure S20. Free energy of dimer formation. Calculated free energy of dimer formation displayed as the values obtained for parallel versus anti-parallel arrangement (A,C) and the difference in free energy between parallel and anti-parallel (B,D) of P0-P10 using residue mediated potentials (A,B) or solvent-mediated potentials (C,D). The blue shaded areas represent ±1kT at 300K.

AlphaFold2 predictions

P0-P10 were also subjected to structural predictions using AlphaFold2,[56] to test whether this software can reproduce the experimentally observed behavior. We used "ColabFold v1.5.3: AlphaFold2 using MMseqs2" on Github. For each peptide, we made five predictions for systems containing between 2-50 chains. In each case, we counted the number of predictions resulting in parallel, anti-parallel and mixed arrangements. Thereby we can answer the question of how many chains are needed in the query for the prediction to be dominated by a parallel arrangement. Figure S21A shows for each peptide how many chains are needed for 1, 2, 3, 4 or 5 (out of 5) predictions to result in parallel arrangement. Figure S21B shows how many chains are needed for 4 (out of 5) predictions to result in a parallel arrangement versus peptide (P0-P9). The number of chains required roughly grows with chain length. We note that an exponential decay can be fitted to the number of chains required versus the number of residues removed relative to P0 (Figure S21B). P1 and P3 are exceptions to this trend with fewer chains required, whereas P2 requires a larger number of chains for parallel predictions to dominate. For peptide P10, AlpaFold2 failed to predict any clusters, therefore no data are included for this peptide in Figure S21.

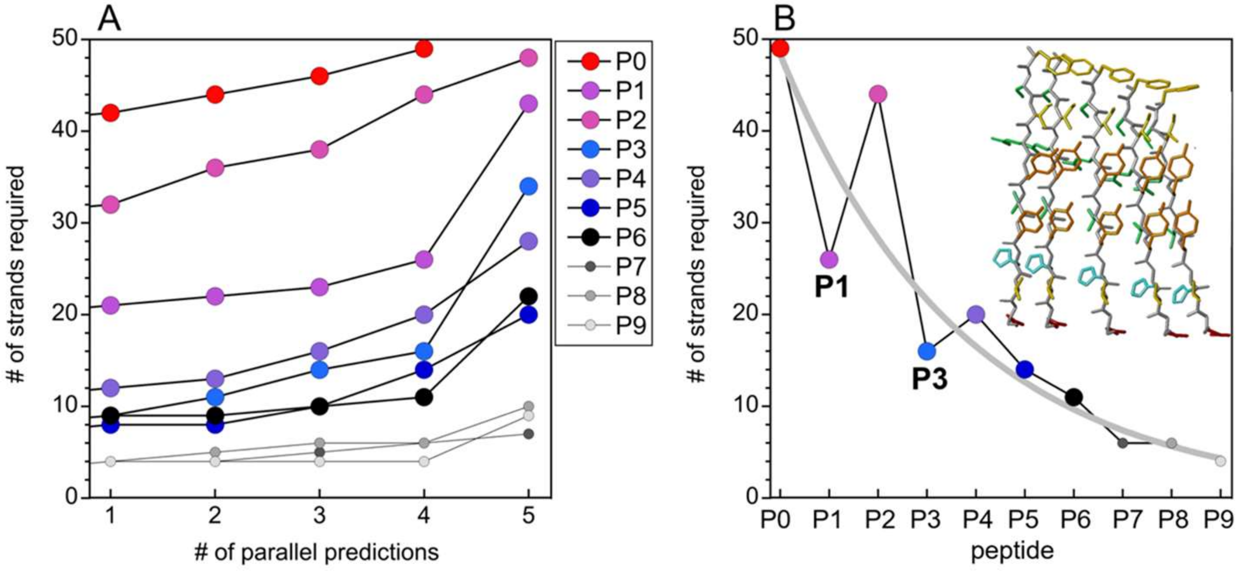

Figure S21. AlphaFold2 predictions of systems of hydrogel peptides. **A**) The number of chains required in the system to predict parallel arrangement in 1, 2, 3, 4 or 5 out of 5 trials shown for P0-P9, with color codes for the peptides indicated to the right. **B**) The number of chains required in the system to predict parallel arrangement in 4 out of 5 trials versus peptide. The grey line is a fitted exponential decay to the number of chains required versus the number of removed residues from P0. The inset shows five chains from a prediction of 50 chains of peptide P3 with side chains colored yellow for F, I and V, orange for Y, green for S, Q, T, cyan for H, and red for D.

Calculation for out-of-registry arrangements

Using the solvent-mediated potentials,[55] we also calculated the free energies for pair-wise arrangements for out-of-register parallel and the two possible out-of-register antiparallel arrangements. For all peptides, except P1, this does not identify any alternative stable structure.

However, the difference between this out-of-register antiparallel arrangement and the parallel in-register arrangement is much smaller than 1 kT, so it is not impossible for these two arrangements to co-exist.

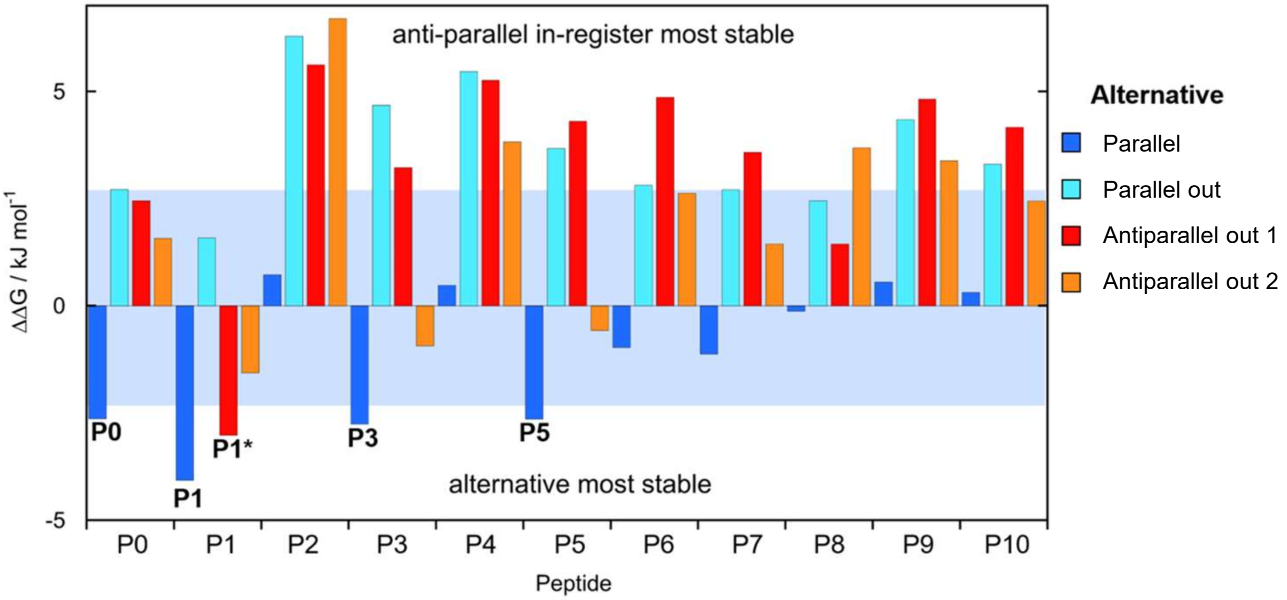

Figure S22. Differences in free energy of dimer formation. The free energy of dimer formation calculated using solvent-mediated potentials displayed as the values obtained for each alternative minus that of the anti-parallel in-register arrangement (AP). The free energy difference is thus plotted relative to AP. For each peptide, the four alternatives displayed are in-register parallel (P, blue), one residue out-of-register parallel (cyan), one residue out-of-register anti-parallel (red), the second possible one residue out-of-register anti-parallel (orange). Upwards bars mean that AP is most stable and down-ward bars that the alternative is most stable. The blue shaded areas represent ±1kT at 300K.

FTIR Spectroscopy

Part D. Phe^{CN} mutants of P0

nitrile stretch region (2200-2270)

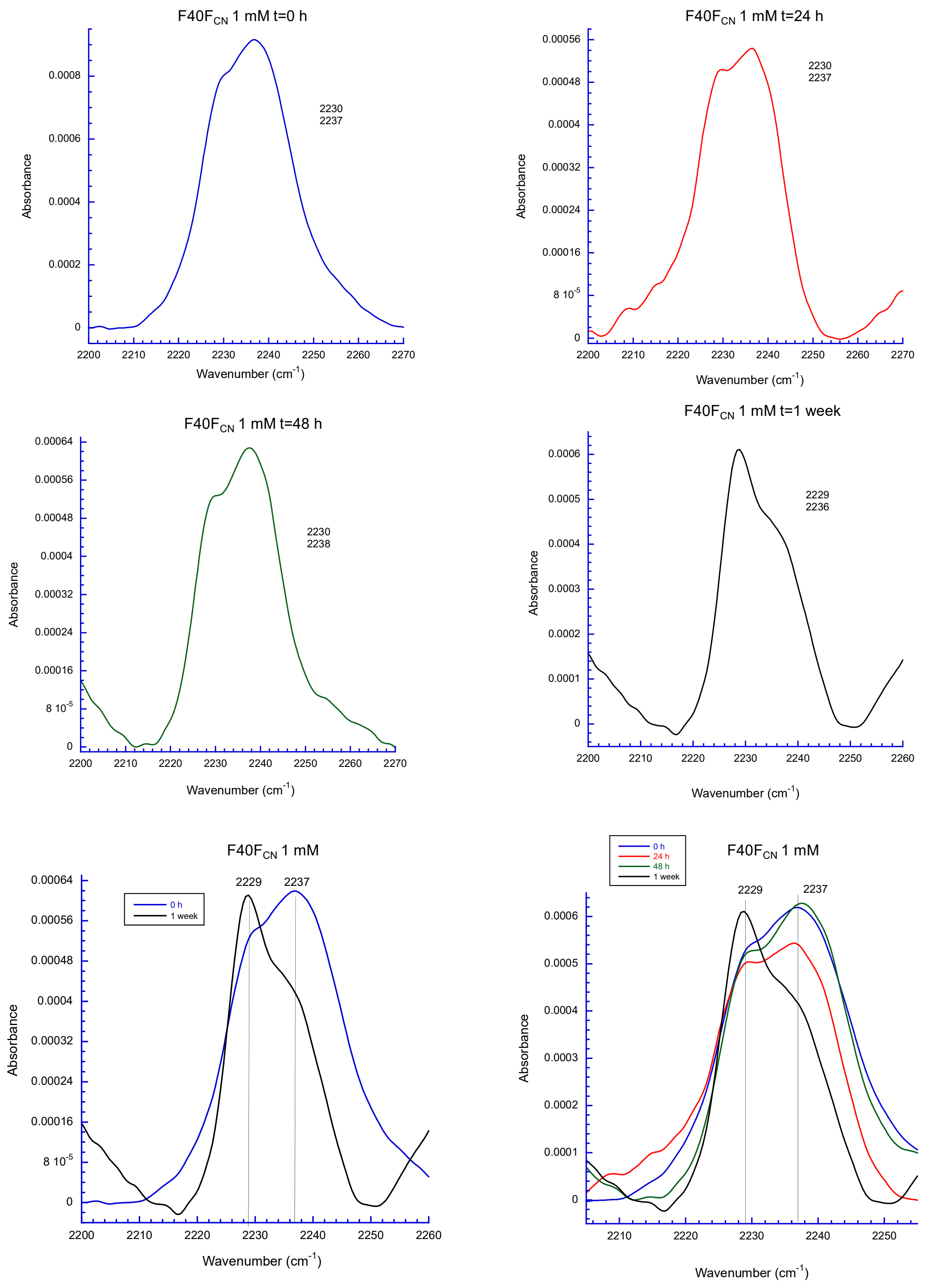

Figure S23. Nitrile region of the FTIR spectra of P0 F40F$_{CN}$ (1.0 mM in ddH_2O) at pH 8.0, incubated at ambient temperature, with samples removed at 0 h, 24 h, 48 h and 1 week (top 4 panels). Overlayed spectra for comparison (bottom 2 panels).

Table S1. spectral data of P0 F40F_{CN} at 1.0 mM dissolved in H_2O at time points t=0, 24, and 48 h, and 1 week, with time point: mode, mean, standard deviation, and width at half height of the nitrile signal.

**FTIR data for F40F_{CN} at 1 mM H_2O (2150 – 2300 cm^{-1})**				
Time Point	Mode (cm^{-1})¶	Mean (cm^{-1})§	Standard Deviation (cm^{-1})	Width at half height (cm^{-1})
t=0 h	2236.58	2235.28	8.66709	18.3200
t=24 h	2236.10	2232.00	9.46288	20.7314
t=48 h	2237.55	2236.94	4.54346	19.2850
t=1 week	2228.87	2232.05	7.89006	17.8387

¶Frequency with the greatest population; §Average frequency within the nitrile peak

amide C=O stretch region (1550-1700)

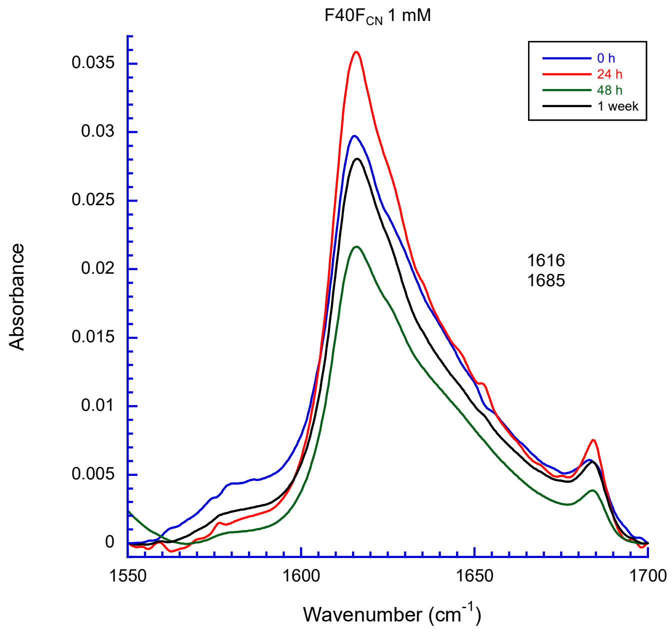

Figure S24. Overlay of the amide I region of the FTIR spectra of P0 F40F_{CN} (1.0 mM in ddH_2O) at pH 8.0, incubated at ambient temperature, with samples removed at 0 h, 24 h, 48 h and 1 w.

Summary

- At 1.0 mM, the cyano group in residue F40F_{CN} becomes more embedded over time (2237 to 2229 cm^{-1}).
- The arrangement of antiparallel strands within the fibril sheet remains intact.
- Mutating F to F_{CN} does not perturb peptide self-assembly.

nitrile stretch region (2200-2270)

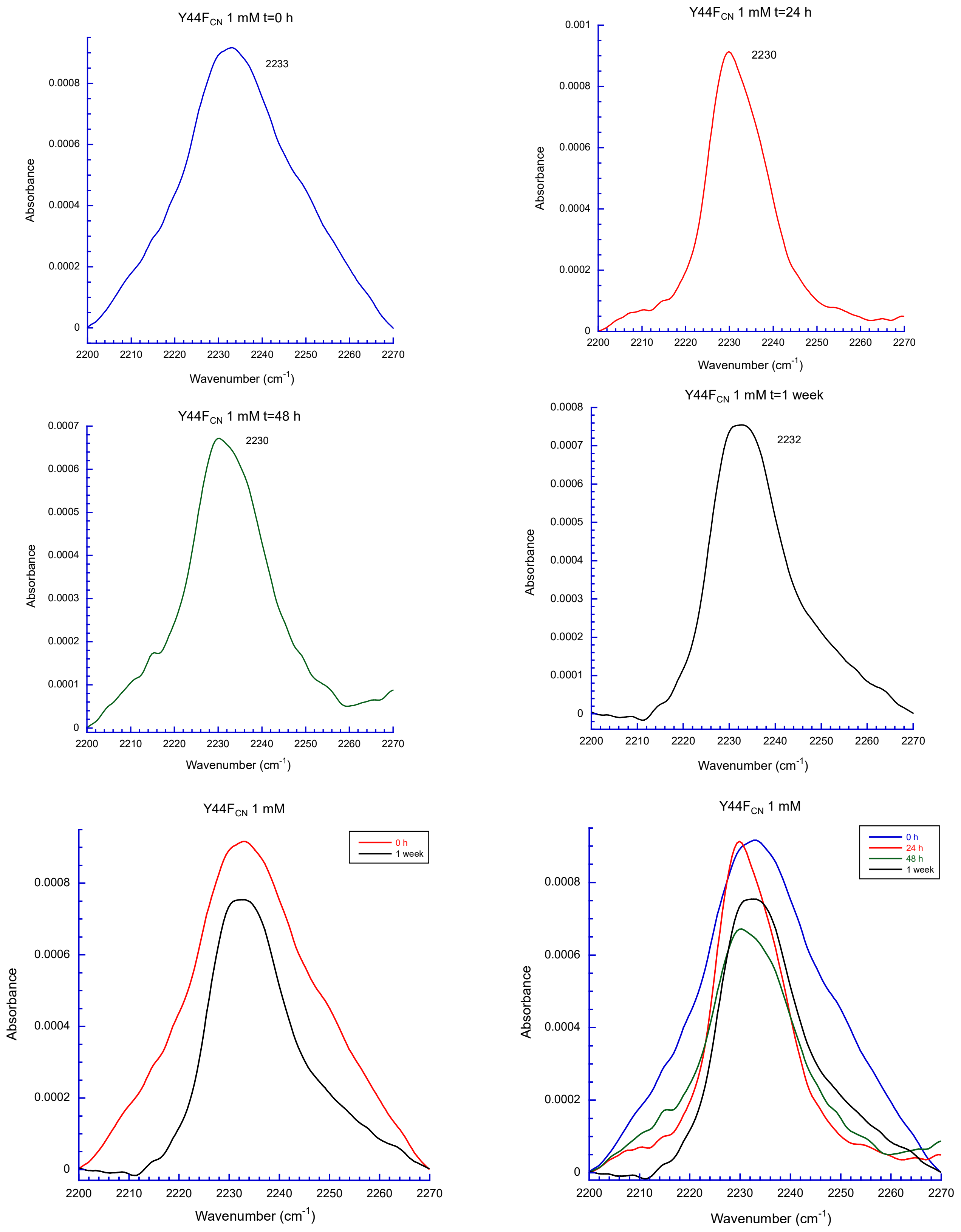

Figure S25. Nitrile region of FTIR spectra of P0 Y44F$_{CN}$ (1.0 mM in ddH_2O) at pH 8.0, incubated at ambient temperature, with samples removed at 0 h, 24 h, 48 h and 1 w (top 4 panels). Overlayed spectra for comparison (bottom 2 panels).

Table S2. IR spectral data of P0 Y44F_{CN} at 1.0 mM dissolved in H_2O at time points t=0, 24, and 48 h, and 1 week, with time point: mode, mean, standard deviation, and width at half height of the nitrile signal.

FTIR data for Y44F_{CN} at 1 mM H_2O (2150 – 2300 cm^{-1})				
Time Point	Mode (cm^{-1})¶	Mean (cm^{-1})§	Standard Deviation (cm^{-1})	Width at Half Max (cm^{-1})
t=0 h	2232.73	2233.12	9.57286	19.2850
t=24 h	2229.83	2232.43	6.91092	14.4638
t=48 h	2230.32	2232.47	8.87285	17.3565
t=1 week	N/A	N/A	N/A	N/A

¶Frequency with the greatest population; §Average frequency within the nitrile peak

amide C=O stretch region (1550-1700)

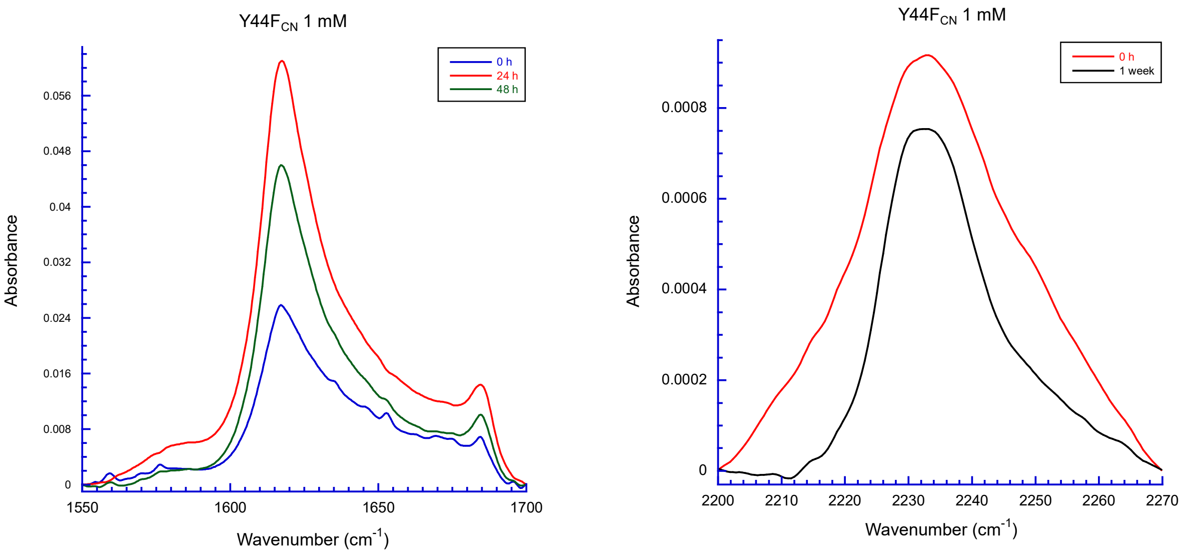

Figure S26. Left: overlay of the amide I region of the FTIR spectra of P0 Y44F_{CN} (1.0 mM in ddH_2O) at pH 8.0, incubated at ambient temperature, with samples removed at 0, 24, and 48 h. Right: overlay of FTIR spectra in the nitrile region at time 0 h and 1 w.

Summary

- At 1.0 mM, the cyano group in residue Y44F_{CN} is more embedded compared to in F40F_{CN} already from the onset of hydrogelation and fibril formation, as indicated by the signal going from 2232 to 2230 cm^{-1} over the course of one week.
- The arrangement of antiparallel strands within the fibril sheet remains constant.

nitrile stretch region (2200-2270)

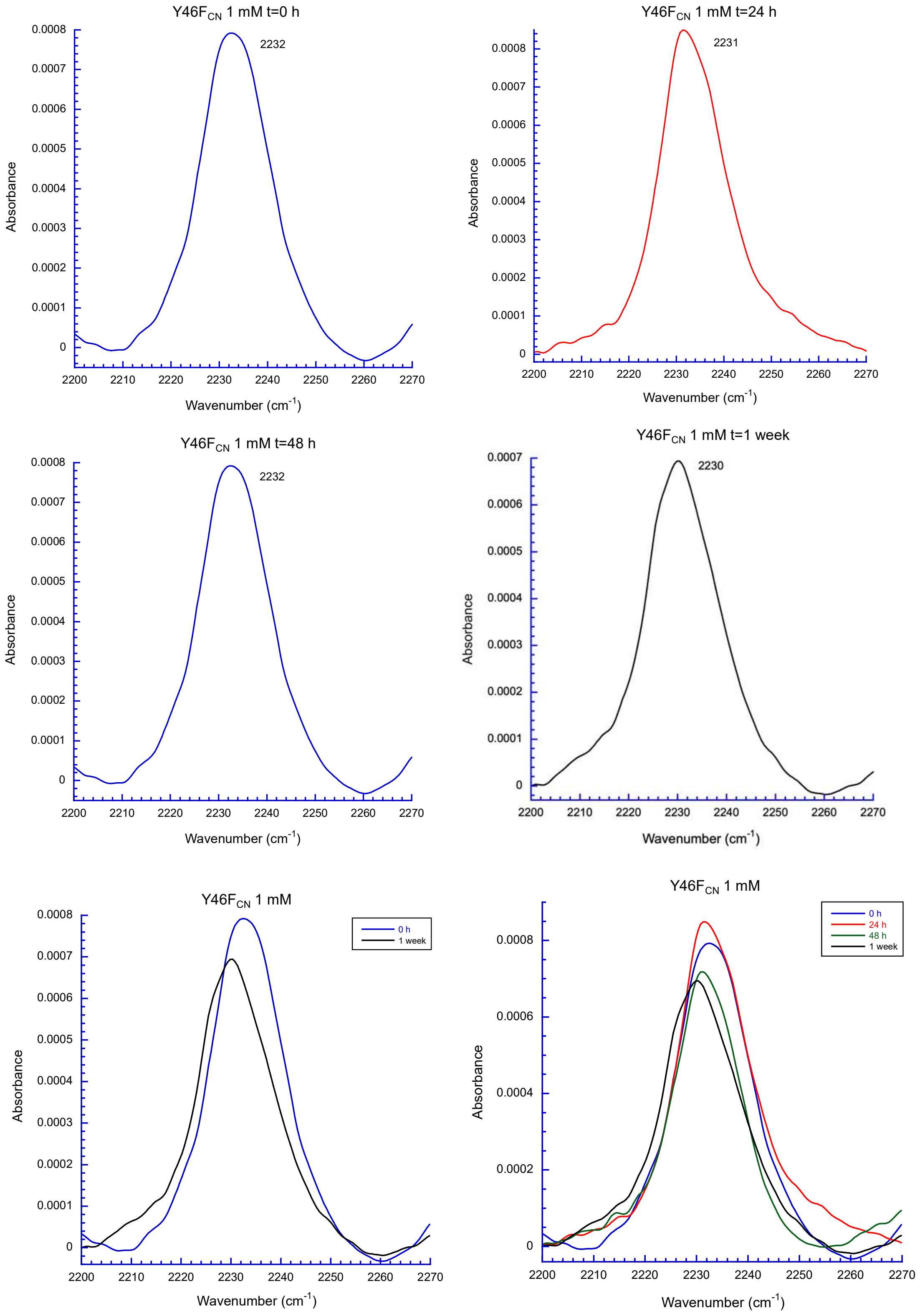

Figure S27. Nitrile region: FTIR spectra of P0 Y46F$_{CN}$ (1.0 mM in ddH$_2$O) at pH 8.0, incubated at ambient temperature, with samples removed at the times indicated, at 0 h, 24 h, 48 h and 1 w (top 4 panels). Overlayed spectra for comparison (bottom 2 panels).

Table S3. IR spectral data of P0 Y46F$_{CN}$ at 1.0 mM dissolved in H_2O at time points t=0, 24, and 48 h, and 1 week, with time point: mode, mean, standard deviation, and width at half height of the nitrile signal.

FTIR data for Y46F$_{CN}$ at 1 mM H_2O (2150 – 2300 cm^{-1})				
Time Point	Mode (cm^{-1})¶	Mean (cm^{-1})§	Standard Deviation (cm^{-1})	Width at Half Max (cm^{-1})
t=0 h	2232.25	2233.03	9.40104	17.8387
t=24 h	2231.28	2233.80	7.08975	14.4638
t=48 h	2231.28	2231.12	8.08644	16.4280
t=1 week	2230.32	2231.40	9.56544	17.3565

¶Frequency with the greatest population; §Average frequency within the nitrile peak

amide C=O stretch region (1550-1700)

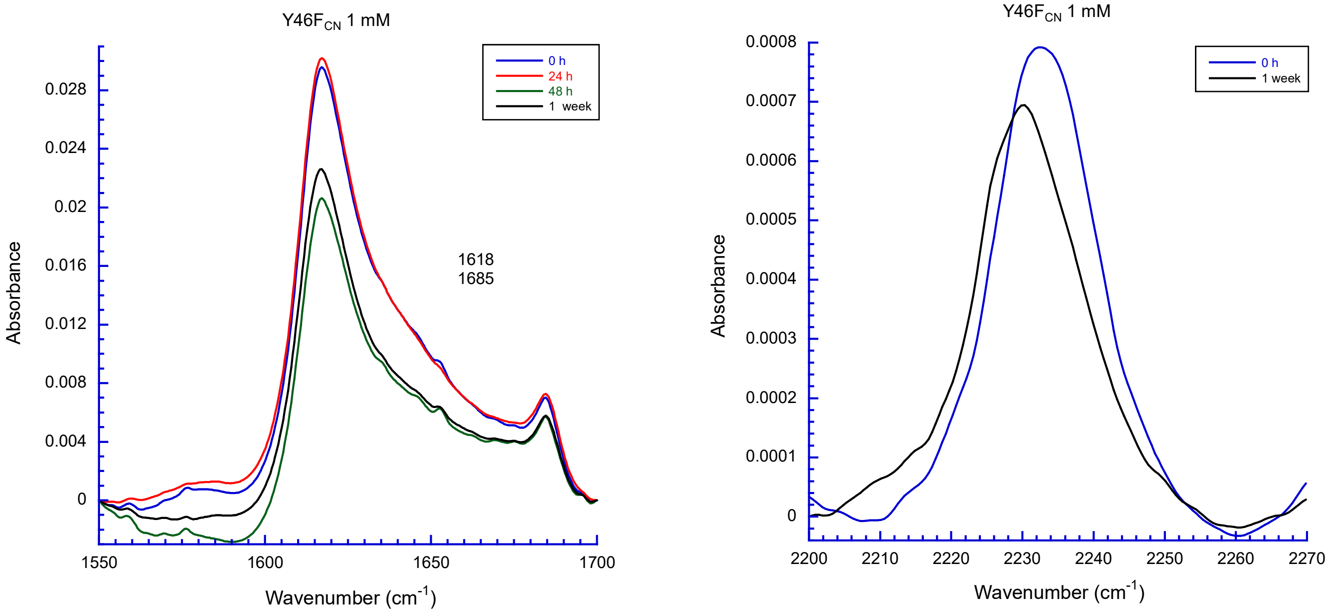

Figure S28. Left: overlay of the amide I region of the FTIR spectra of P0 Y46F$_{CN}$ (1.0 mM in ddH_2O) at pH 8.0, incubated at ambient temperature, with samples removed at 0, 24, and 48 h. Right: overlay of the nitrile region of FTIR spectra at time 0 h and 1 w.

Summary

- Similarly to P0 Y44Fcn, at 1.0 mM, the cyano group in residue Y46Fcn is more embedded compared to F40Fcn already from the start of hydrogelation and fibril formation, as indicated by the signal going from 2232 to 2230 cm^{-1} over the course of one week.
- The arrangement of antiparallel strands within the fibril sheet remains constant.

Part E. *cryo*-TEM images and SAXS/WAXS data for P0-P6

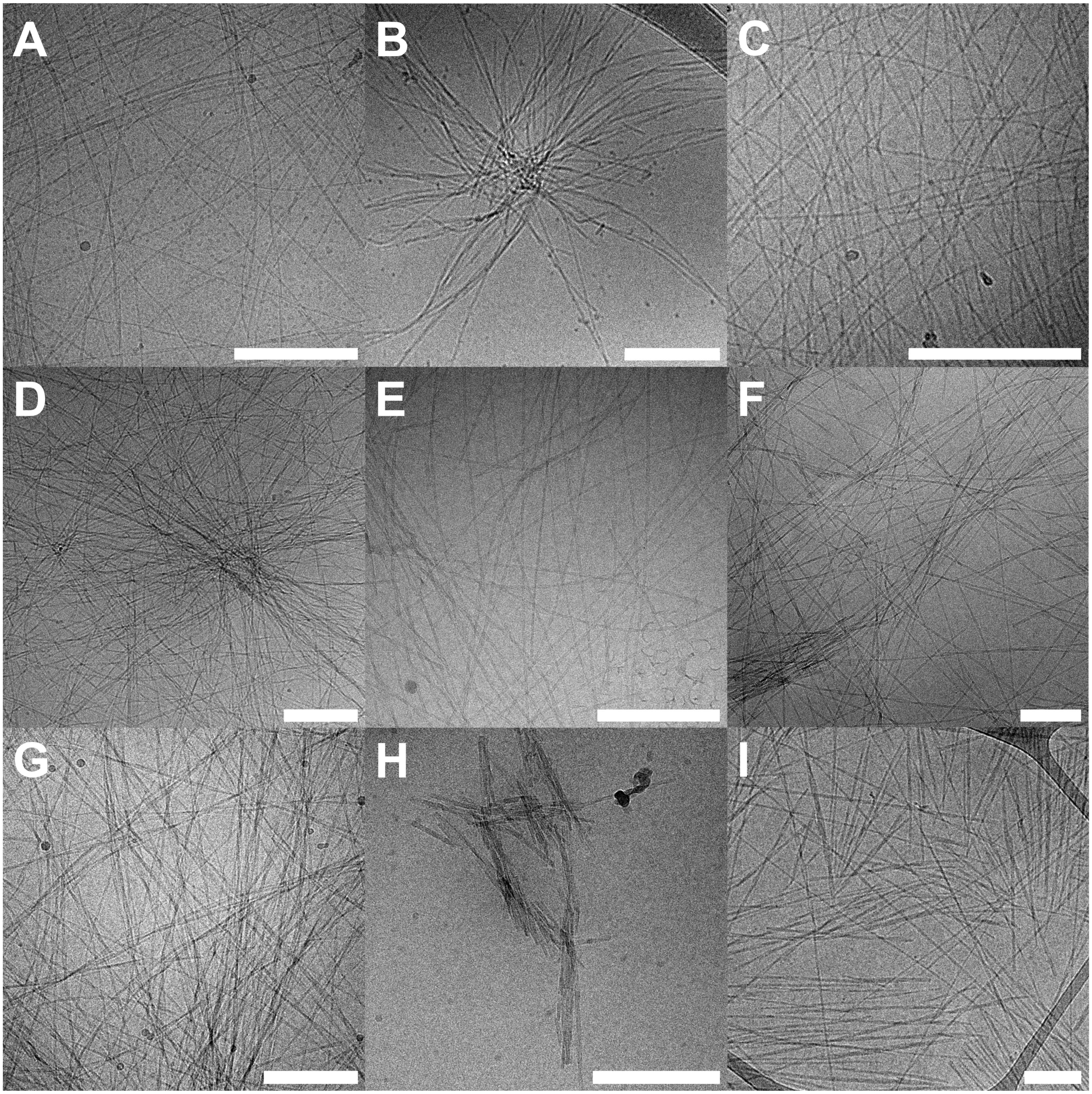

Figure S29. *Cryo*-TEM images of P0-P6 (for additional images, see Figure 7). (A) P0, (B) P1, (C) P2, (D) P2, (E) P3, (F) P4, (G) P5, top layer of phase-separated sample, (H) P5, bottom layer of phase-separated sample, and (I) P6. The images were taken after 2 days of incubation at 21 °C in water at pH 8.0, except E, which was taken after 1 week of incubation. Most samples were imaged at a concentration of 2.0 mM except panels A, B, and D, which were imaged at 100 μM. The white scale bar in all images represents 200 nm.

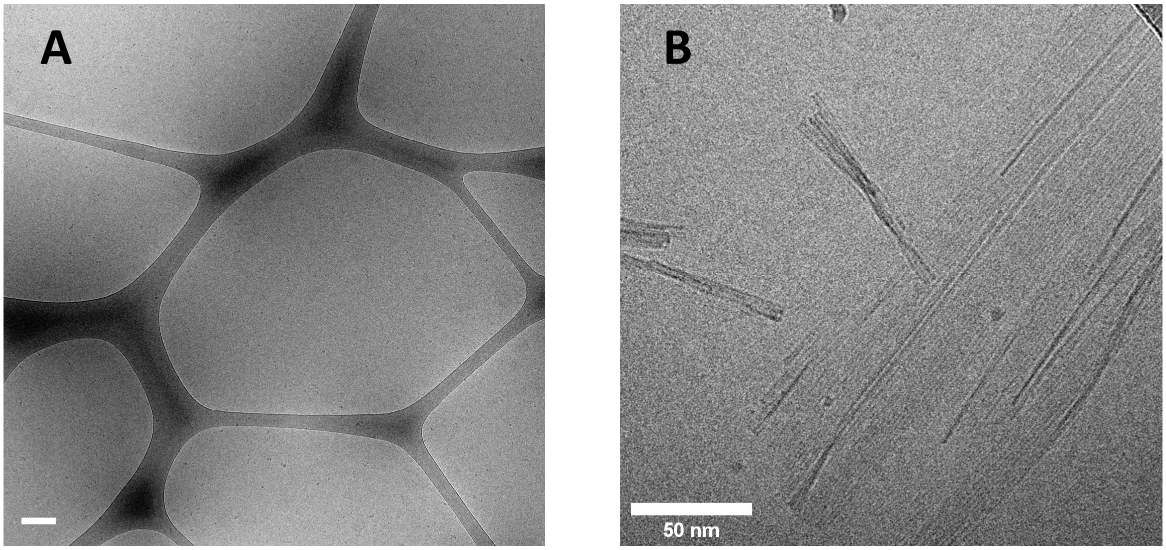

Figure S30. (A) A representative *cryo*-TEM image of P7 after 1 week of incubation at ambient temperature. No nanofibers were observed in the sample, consistent with FTIR and SAXS data for this sequence. Scale bar = 200 nm. **(B)** *Cryo-TEM* image of P5 after 3 hours of incubation at ambient temperature shows aligned nanofibers that form sheet-like structures. Scale bar = 50 nm.

Table S4. Parameters used in the elliptical cylinder scattering model.

Sample	Scale	Background [cm^{-1}]	Minor Radius [nm]	Axial ratio	Length [nm]	Peptide Scattering Length Density (Å^{-2})	Solvent Scattering Length Density (Å^{-2})	Fitting Error (χ^2)
P0 day 7	1.0 10^{-3}	6.0 10^{-4}	1.3	2.8	2000	1.3 10^{-5}	9.5 10^{-6}	8.3 10^{-2}
P1 day 7	6.5 10^{-4}	4.0 10^{-3}	1.5	2.5	2000	1.3 10^{-5}	9.5 10^{-6}	2.2 10^{-4}
P2 day 7	7.0 10^{-4}	5.0 10^{-4}	1.8	3.5	2000	1.3 10^{-5}	9.5 10^{-6}	4.5 10^{-3}
P3 day 7	7.0 10^{-4}	3.0 10^{-4}	1.8	3.2	2000	1.3 10^{-5}	9.5 10^{-6}	1.2 10^{-1}
P4 day 7	2.9 10^{-4}	3.0 10^{-4}	3.0	2.1	2000	1.3 10^{-5}	9.5 10^{-6}	1.8 10^{-2}
P5 day 7	1.3 10^{-4}	3.5 10^{-4}	3.3	1.9	2000	1.3 10^{-5}	9.5 10^{-6}	1.5 10^{-4}
P6 day 7	3.5 10^{-4}	4.5 10^{-4}	3.1	1.9	2000	1.3 10^{-5}	9.5 10^{-6}	4.5 10^{-3}

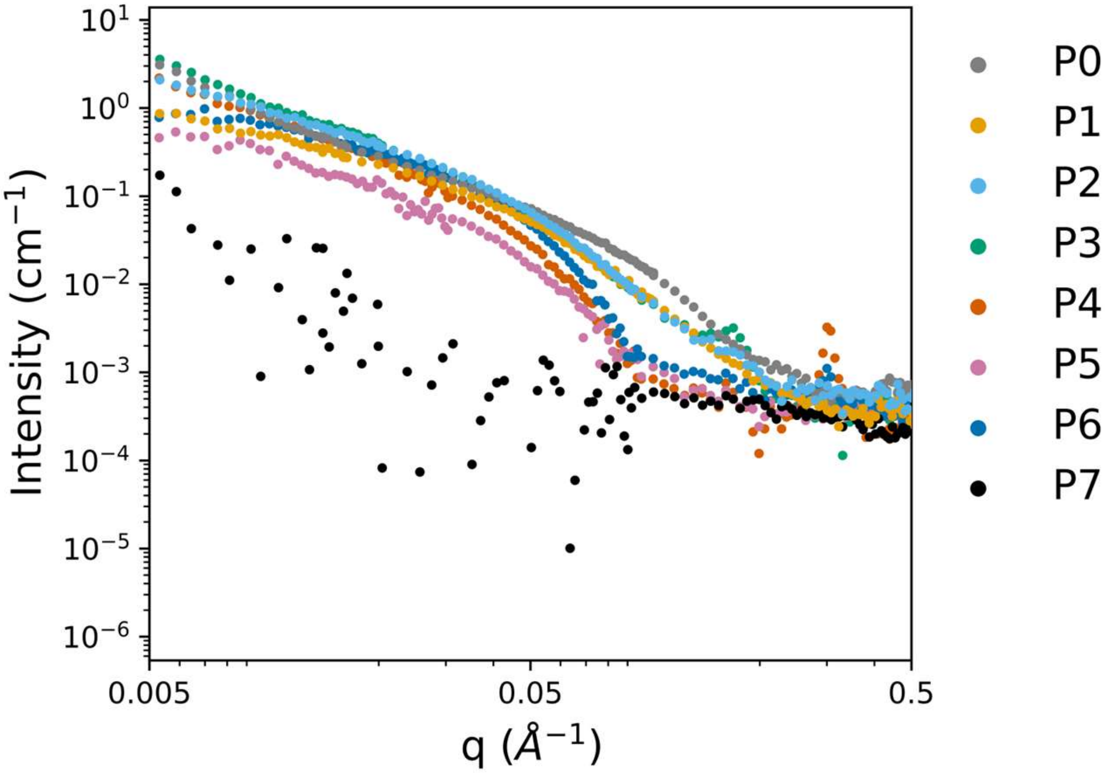

Figure S31. X-ray scattering data for P0-P7 on an absolute scattering intensity scale. A major change in the shape of the scattering curves is observed between P3 and P4, which corresponds to a dramatic increase in the fibril cross-sectional area.

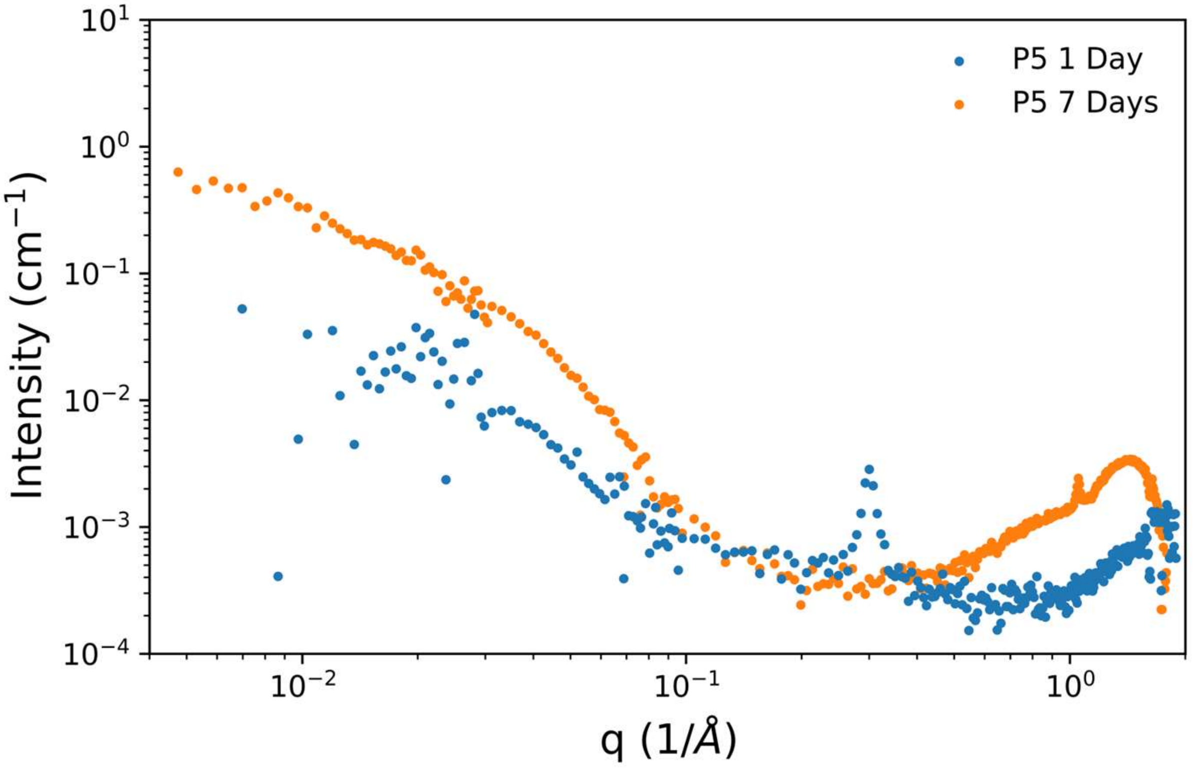

Figure S32. X-ray scattering curves of 2.0 mM P5 at day 1 and 7 days after hydrogel preparation show that at day 1 P5 has a strong reflection at 0.2 Å^{-1}. The increase in scattering intensity over the week of incubation is indicative of a progressive increase in fibril formation over the course of the experiment.
